# Initial Psychometric Evaluation of the Inventory of Parent and Peer Attachment in Greek Adolescents

**DOI:** 10.3390/ejihpe16090134

**Published:** 2026-09-02

**Authors:** Georgios Giannakopoulos, Aikaterini Fostini, Renati Marinou, Varvara Salavou

**Affiliations:** Department of Child and Adolescent Psychiatry, School of Medicine, National and Kapodistrian University of Athens, Aghia Sophia Children’s Hospital, 115 27 Athens, Greece; fostinik@hotmail.com (A.F.); renatimarinou@gmail.com (R.M.); varvsalavou@med.uoa.gr (V.S.)

**Keywords:** Inventory of Parent and Peer Attachment, perceived relational security, parent-adolescent relationship quality, peer relationship quality, psychometric evaluation, Greece, SDQ, reliability, structural validity

## Abstract

Adolescents’ perceived security in relationships with parents and peers relates to psychosocial adjustment, yet the psychometric performance of the Inventory of Parent and Peer Attachment (IPPA) has not been examined in Greece. This study evaluated the Greek IPPA in a non-probability sample of 432 adolescents aged 12–16 years from five schools in Attica (Greece). Internal consistency, item-level analyses, floor and ceiling effects, and one-factor, two-factor, correlated three-factor, and bifactor models were examined using ordinal WLSMV estimation. Relations with Strengths and Difficulties Questionnaire scores were assessed. Parent Total Security and Peer Total Security showed high internal consistency (α = 0.94 and 0.93, respectively; ω = 0.94 for both). The correlated three-factor model showed acceptable fit for the parent form and weaker fit for the peer form, while Trust and Communication were highly correlated in both forms. Bifactor indices indicated substantial general-factor saturation, although the specific Trust factors were poorly defined. Higher total security scores were associated with fewer psychosocial difficulties. Overall, the parent and peer total scores appear more defensible than the separate subscales for preliminary, group-level descriptive use in similar samples, whereas subscale interpretation, particularly distinctions between Trust and Communication, should remain cautious. These findings require independent replication and do not support individual clinical decision-making or the identification of attachment classifications, attachment disorders, or disorganization.

## 1. Introduction

Attachment theory provides a major developmental framework for understanding how early and continuing relationships shape emotion regulation, interpersonal expectations, and psychosocial adaptation. Bowlby conceptualized attachment as a biologically grounded behavioral system through which children seek proximity to protective figures under conditions of threat or distress (Bowlby, 1969, 1973, 1980). Ainsworth and colleagues operationalized individual differences in attachment security in infancy, while later representational approaches emphasized internal working models of self and others that organize expectations about availability, trust, and responsiveness across development (Ainsworth et al., 1978; Bretherton, 1992; Main et al., 1985).

Although the attachment system originates in early caregiving relationships, adolescence is a particularly important developmental period for reassessing attachment processes. Adolescents increasingly seek autonomy while continuing to rely on parents as secure-base figures, especially under conditions of high stress or uncertainty (Allen, 2008; Allen & Tan, 2016; Moretti & Peled, 2004). At the same time, peers become increasingly salient sources of intimacy, support, identity exploration, social belonging, and emotional disclosure (Brown & Bakken, 2011; Furman & Buhrmester, 1992; Gorrese & Ruggieri, 2012; Laible et al., 2000). This developmental reorganization does not mean that peers replace parents. Rather, attachment-related functions become distributed across a broader relational network, with parents and peers often contributing in distinct but complementary ways (Allen & Tan, 2016; Fraley & Davis, 1997; Gorrese & Ruggieri, 2012; Nickerson & Nagle, 2005).

Valid assessment of attachment during adolescence is therefore essential for both research and clinical practice. However, attachment measurement in middle childhood and adolescence remains methodologically challenging. In contrast to infancy, where observational paradigms such as the Strange Situation have historically dominated, and adulthood, where both interview-based and self-report instruments are widely used, adolescence requires measures that capture adolescents’ own representations of ongoing relationships with multiple attachment figures (Crowell et al., 2016; Hesse, 2016; Muzi et al., 2022). A systematic review using COSMIN criteria concluded that only a limited number of attachment measures for middle childhood and adolescence have adequate psychometric evidence, and that the Inventory of Parent and Peer Attachment (IPPA) has the strongest evidence among self-report instruments, despite continuing limitations in structural validity and cross-cultural evidence (Jewell et al., 2019).

The IPPA was developed by Armsden and Greenberg (1987) to assess adolescents’ perceived attachment security toward parents and peers. It is not a categorical attachment-style measure and does not classify adolescents as secure, avoidant, ambivalent, or disorganized. Instead, it assesses perceived relationship quality dimensionally across three theoretically derived domains: Trust, Communication, and Alienation (Armsden & Greenberg, 1987). Trust refers to perceived reliability, acceptance, mutual respect, and emotional availability. Communication refers to openness and responsiveness in discussing worries, feelings, and personal concerns. Alienation refers to emotional distance, anger, loneliness, or perceived lack of belonging.

More broadly, self-report measures such as the IPPA capture adolescents’ explicit perceptions of attachment-related quality in their current relationships and should be distinguished from representational and observational approaches to attachment assessment. Interview-based measures assess attachment representations partly through the organization and coherence of attachment-related narratives, whereas observational approaches focus on behavior within attachment-relevant interactions. These methods address related but non-equivalent aspects of attachment and may show only partial convergence across populations (Madigan et al., 2016; Muzi et al., 2022). Accordingly, the IPPA is most pertinent when the research question concerns adolescents’ own perceptions of trust, communication, and alienation in parent and peer relationships; it should not be treated as a substitute for interview- or observation-based assessment of attachment organization or classification.

The IPPA has been widely adapted and used across cultural contexts, but its factor structure remains debated. Some studies support the original three-factor model, whereas others support one-factor, two-factor, or modified three-factor solutions (Demetriou et al., 2022; Gullone & Robinson, 2005; Habibi Asgarabad et al., 2025; Marini et al., 2025; Maya et al., 2023; Pace et al., 2011; Tohme et al., 2024). The Italian validation by Pace et al. compared one-factor, two-factor, and three-factor models in 1059 adolescents and found that the three-factor model showed the best fit, although the three dimensions were strongly interrelated (Pace et al., 2011). The Cypriot validation in Greek-speaking clinical and non-clinical adolescents supported the original three-dimensional structure for both parent and peer forms and found meaningful associations with adolescent emotional and behavioral problems and family functioning variables (Demetriou et al., 2022). By contrast, studies in clinical or at-risk samples have sometimes found weaker support for the peer form. In Spanish adolescents with behavioral problems, the 25-item three-factor structure was confirmed for mother and father forms but not for the peer version (Maya et al., 2023). Similarly, the Arabic IPPA-R validation in Lebanese adolescents found that a modified three-factor structure and item reduction were needed to achieve satisfactory psychometric performance (Tohme et al., 2024).

The peer form appears to be the most psychometrically complex component of the IPPA. There are several plausible reasons for this. First, adolescent friendships are developmentally fluid, and the peer referent may vary across respondents, from a best friend to a broader friendship group (Furman & Buhrmester, 1992; Gorrese & Ruggieri, 2012). Second, Trust and Communication may be less separable in peer relationships than in parent relationships. Adolescents may experience a peer as trustworthy precisely because they can communicate openly with that peer, and open communication may itself be the central behavioral expression of trust (Habibi Asgarabad et al., 2025; Pace et al., 2011; Tohme et al., 2024). Third, Alienation in peer relationships may capture multiple phenomena, including loneliness, rejection, anger, social exclusion, unmet contact needs, or social anxiety (Habibi Asgarabad et al., 2025; Laible et al., 2000; Marini et al., 2025). Fourth, contemporary digital communication may require a more cautious interpretation of peer items referring to contact and closeness. Recent adolescent research suggests that smartphones and online platforms are now embedded in adolescents’ social communication and peer-related functioning (Anbumalar & Binu Sahayam, 2026; Marini et al., 2025). Therefore, a desire for more frequent contact with friends may not necessarily indicate insecurity or alienation; in some adolescents, it may also reflect normative connectedness, peer salience, or digitally mediated social belonging (Demetriou et al., 2022).

Attachment security is also clinically meaningful. Secure parent and peer attachment have been associated with fewer internalizing and externalizing difficulties, lower aggression, better emotional regulation, higher self-esteem, stronger social competence, and better overall well-being (Brumariu & Kerns, 2010; Fearon et al., 2010; Groh et al., 2012; Kobak et al., 2015; Madigan et al., 2016; Oldfield et al., 2016). Parent attachment often shows broad associations with emotional and behavioral adjustment, while peer attachment may be particularly relevant to loneliness, peer problems, body dissatisfaction, school belonging, and prosocial behavior (Chan et al., 2025; Delgado et al., 2022; Gorrese & Ruggieri, 2012; Oldfield et al., 2016; Pallini et al., 2014). Recent IPPA-based studies continue to show that attachment relationships are implicated in diverse adolescent and emerging-adult outcomes, including depression, psychotic-like experiences, smartphone addiction, eating-related symptoms, and adjustment in families affected by chronic illness (Anbumalar & Binu Sahayam, 2026; Çam Ray et al., 2025; H. Hu et al., 2026; Lavorgna et al., 2026; K. Li et al., 2026; Qin et al., 2025). These findings support the relevance of validating the IPPA in culturally specific adolescent populations.

A Greek validation is needed for several reasons. First, Greek adolescent research requires developmentally appropriate tools that assess adolescents’ perceived relational security, not only symptoms. Second, although a Greek-speaking Cypriot validation exists, Greece and Cyprus differ in educational context, sampling frame, and sociocultural conditions; local psychometric data are therefore necessary before using the instrument confidently in Greek school-based studies (Demetriou et al., 2022). Third, the IPPA has potential value in school mental health research as a descriptive measure of adolescents’ perceived trust, communication, and alienation, particularly when studied alongside emotional and behavioral problem measures. Fourth, describing the score distribution in a local sample may facilitate comparison across future Greek studies. Such descriptive information should not, however, be treated as population reference values or norms without representative sampling and evidence that the scores function comparably across relevant demographic groups (American Educational Research Association et al., 2014; Kane, 2013).

Three hypothesis-driven expectations were specified. First, previous psychometric studies of the IPPA have generally reported strong internal consistency for the overall parent and peer attachment scales (Armsden & Greenberg, 1987; Pace et al., 2011). On this basis, we expected Parent Total Security and Peer Total Security to show excellent internal consistency, operationalized as Cronbach’s α and McDonald’s ω values of at least 0.90; coefficients of at least 0.70 were considered adequate and values of at least 0.80 good (Stefana et al., 2025).

Second, Pace et al. (2011), in a sample of 1059 Italian adolescents, found that the original correlated three-factor model provided a better fit than one- and two-factor alternatives, although Trust, Communication, and Alienation were strongly interrelated. Demetriou et al. (2022) also supported the original three-dimensional structure in Greek-speaking Cypriot adolescents, whereas studies by Maya et al. (2023) and Tohme et al. (2024) indicated greater structural difficulties for the peer form. We therefore expected the original correlated three-factor model to provide a better representation of the parent and peer forms than simpler one- and two-factor alternatives, while anticipating substantial overlap between Trust and Communication, particularly in the peer form. For discriminant validity, we nevertheless expected Trust, Communication, and Alienation to remain statistically distinguishable rather than being perfectly correlated, while recognizing that strong Trust–Communication correlations could indicate limited practical differentiation between these dimensions. This expectation was specified explicitly in line with the hypothesis-driven approach to construct-validity evaluation recommended by Wetzel and Killisch (2025).

The correlated three-factor model was therefore designated as the primary structural model. Structural validity was evaluated from its relative performance against the simpler alternatives together with the pattern of robust CFI, TLI, RMSEA, and SRMR indices. Bifactor models were treated as secondary diagnostic models for evaluating the extent to which the total scores represented broad general perceived-security dimensions alongside narrower specific factors.

Third, previous studies have linked parent and peer attachment with adolescent psychosocial adjustment. Demetriou et al. (2022) reported associations of IPPA dimensions with adolescent emotional and behavioral problems, while Oldfield et al. (2016) found associations of parental and peer attachment with adolescent mental health outcomes. Broader evidence also links attachment-related relationship quality with internalizing and externalizing difficulties and peer-related adjustment (Gorrese & Ruggieri, 2012; Madigan et al., 2016). We therefore specified a priori directional expectations that higher Parent Total Security and Peer Total Security scores would correlate negatively with emotional and behavioral difficulties and positively with prosocial behavior, consistent with the hypothesis-driven approach to validity evidence advocated by Wetzel and Killisch (2025). Because the SDQ assesses psychosocial adjustment rather than attachment itself, these associations were considered evidence based on relations with theoretically relevant external variables rather than convergent validity in the narrow sense of agreement between alternative measures of the same construct.

Sex differences, participating school-site differences, associations with parent-reported educational level, occupational status, subjective family economic status and perceived social class, and descriptive sample percentiles were treated as secondary exploratory or descriptive analyses rather than confirmatory hypotheses.

Accordingly, the objective of the present study was to provide an initial psychometric evaluation of the Greek IPPA in a community sample of adolescents aged 12–16 years from Attica (Greece). The primary outcomes were (a) the internal consistency of Parent Total Security and Peer Total Security and (b) the structural validity of the parent and peer forms. A prespecified secondary construct-validity outcome was the pattern of associations between the two total security scores and SDQ indices. Additional planned analyses examined item-level performance, latent-factor distinctiveness, bifactor-derived reliability and dimensionality indices, and secondary exploratory or descriptive differences by sex, school site, and socioeconomic characteristics, as well as descriptive sample percentiles.

## 2. Materials and Methods

### 2.1. Participants

Participants were adolescents enrolled in the three grades of five lower-secondary schools (Gymnasia) in Attica (Greece). The participating school sites formed a non-probability convenience sample. They were recruited through communication with the relevant educational authorities and school principals and were included on the basis of institutional accessibility and willingness to participate. No random or stratified school-selection procedure was used. The available study records identify the five participating school sites but do not provide a complete log of any additional schools that may have been contacted before the final sites were confirmed.

The five participating school sites were located within four Directorates of Secondary Education in Athens. One site was located within each of the A, C, and D Athens Directorates, while two sites belonged to the B Athens Directorate. For confidentiality reasons, individual schools are not named and are reported as School Sites 1 to 5. Accordingly, school-site comparisons should be interpreted as comparisons between the participating schools and not as comparisons between the four Directorates of Secondary Education.

**Figure 1:**
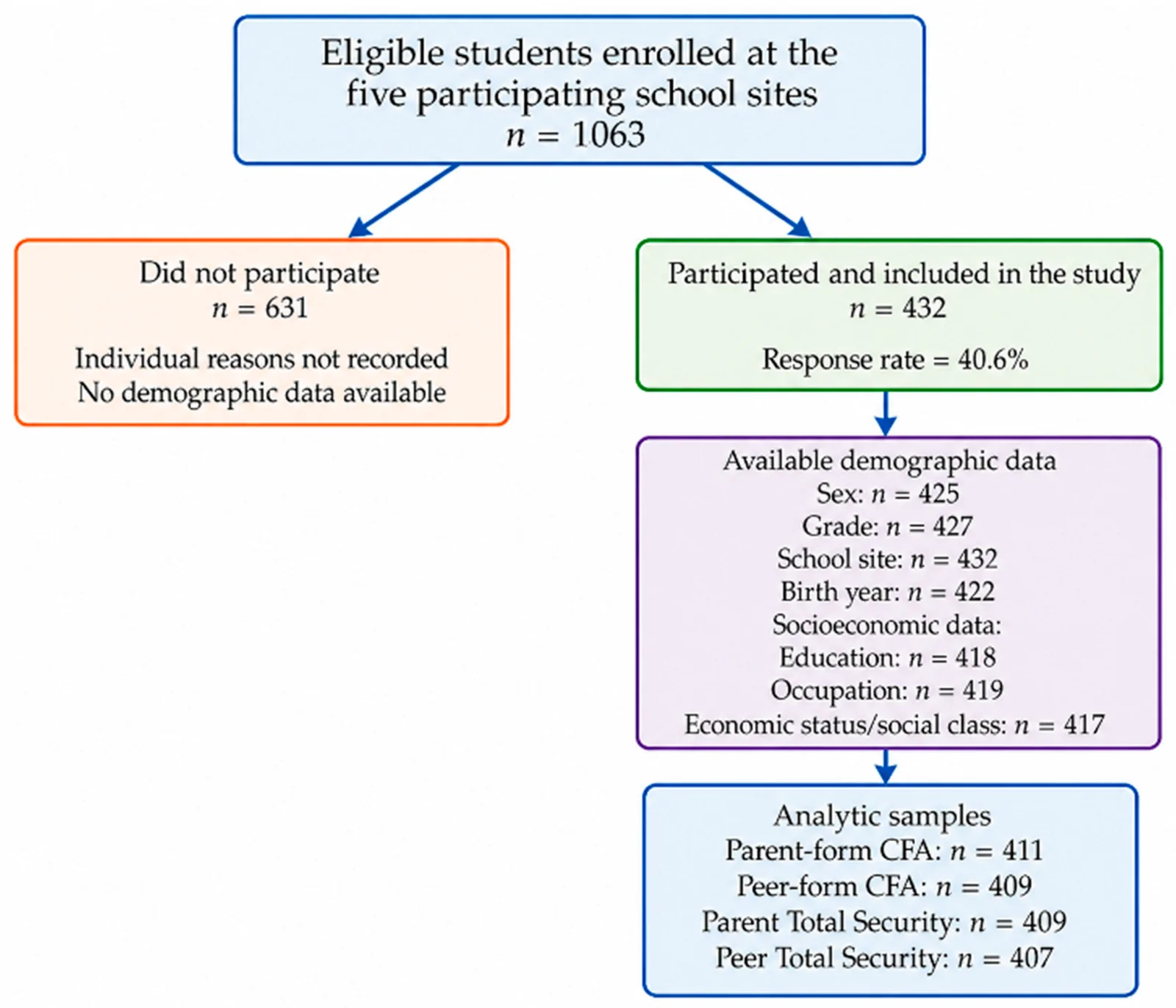
Participant recruitment and analytic flow. Note. CFA = confirmatory factor analysis. The CFA samples included participants with at least one valid item response on the relevant IPPA form. Parent Total Security and Peer Total Security were calculated only when at least 20 of the 25 relevant items had valid responses.

Eligibility criteria were age 12–16 years, enrollment in a lower-secondary school (Gymnasium) in Attica, sufficient understanding and use of the Greek language to complete the questionnaires, written parental or legal-guardian consent, and adolescent assent. No additional exclusion criteria were applied. Across the five school sites, 1063 eligible students were invited. Of these, 432 participated and were included in the study, yielding a student-level response rate of 40.6%. School-specific response rates were 44.5% (89/200) for School Site 1, 58.7% (111/189) for School Site 2, 30.7% (75/244) for School Site 3, 29.6% (80/270) for School Site 4, and 48.1% (77/160) for School Site 5. Response rates differed significantly across the five participating school sites, χ^2^(4) = 54.08, *p* < 0.001, Cramér’s *V* = 0.23. The remaining 631 eligible students did not participate. Individual reasons for non-participation were not recorded systematically, and the available data do not permit classification of non-participants according to absence, non-return of parental consent, parental refusal, or adolescent refusal. No demographic or questionnaire information was collected from non-participants; consequently, direct comparisons between participants and non-participants were not possible. Participant flow is shown in Figure 1.

Valid sex data were available for 425 participants, including 212 girls and 213 boys. Valid grade data were available for 427 participants: 163 attended the first grade of Gymnasium, 145 attended the second grade, and 119 attended the third grade. Valid school-site data were available for 432 participants: School Site 1, *n* = 89; School Site 2, *n* = 111; School Site 3, *n* = 75; School Site 4, *n* = 80; and School Site 5, *n* = 77. Valid age data were available for 422 participants. At the March 2026 assessment, participants had a mean age of 13.95 years (*SD* = 0.85; range = 12–16 years). The corresponding birth-year distribution was 2010, *n* = 8; 2011, *n* = 116; 2012, *n* = 143; and 2013, *n* = 155. The study sample was not intended to be nationally representative of Greek adolescents.

Family background information was most commonly provided by mothers. Among participants with valid parent-informant data, 83.0% of informants were mothers and 17.0% were fathers. Most adolescents lived in families in which parents were married. Place of residence was predominantly urban, with most participants living in a large city and approximately one quarter living in suburbs or the outskirts of a large city.

Parent-informant educational level was available for 418 participants, occupational status for 419 participants, and subjective family economic status and perceived social class for 417 participants each. Most parent-informants had completed post-secondary or university education and were employed full-time. Most families were described as having average subjective economic status and as belonging to the middle or upper-middle social class. Participant characteristics are summarized in Table 1.

### 2.2. Procedure

This was a cross-sectional, school-based initial psychometric evaluation of the Greek version of the Inventory of Parent and Peer Attachment (IPPA), with recruitment and data collection conducted in March 2026. To support transparent reporting of the questionnaire-based study procedures, the manuscript was reviewed against the survey-reporting considerations summarized by Turk et al. (2018), including participant recruitment and eligibility, mode of questionnaire administration, response rate, questionnaire completion, missing-data handling, informed consent, and protection of participant privacy. School participation was arranged after communication with the relevant educational authorities and school principals. Information materials and consent forms were distributed to the eligible students through the participating schools. Parents or legal guardians received written information about the aims and procedures of the study and were asked to provide written informed consent. Adolescents whose parents or guardians had provided consent received age-appropriate information and provided assent before questionnaire administration. No non-response questionnaire was administered, and reasons for non-participation were not recorded at the individual-student level.

Questionnaire administration took place during school hours, with permission from the principals of the participating schools to administer the questionnaires during regular class time. A teacher was present in the classroom during questionnaire administration. Questionnaires generally took approximately 15 min to complete, but no fixed time limit was imposed, and adolescents were allowed as much time as they needed. Administration was conducted under conditions that protected the privacy of individual responses. Members of the research team were present throughout the administration to provide clarification or answer questions when needed.

Parents or legal guardians completed a separate family-background and sociodemographic form included with the consent materials. Adolescents completed the Greek IPPA parent and peer forms and the Strengths and Difficulties Questionnaire (SDQ) during the school assessment. To protect confidentiality, each participant was identified only by a numerical study code; names and other directly identifying information were not recorded on the questionnaires or included in the analytic dataset. Study data were securely stored and were accessible to the research team.

### 2.3. Measures

#### 2.3.1. Inventory of Parent and Peer Attachment

The Inventory of Parent and Peer Attachment (IPPA) is a self-report instrument designed to assess adolescents’ perceptions of trust, communication, and alienation in their relationships with parents and peers (Armsden & Greenberg, 1987). It provides dimensional information about perceived attachment-related relationship quality. It does not assess attachment organization through observational or interview-based methods and cannot identify categorical attachment classifications, attachment disorders, or disorganization.

The Greek version used in the present study included the 25-item parent form and the 25-item peer form. In the parent form, the instructions and individual items referred to “parents” collectively (e.g., “my parents”). Adolescents were not instructed to nominate a single parent, primary caregiver, or other adult as the referent, and no specific parent referent was recorded. Items were rated on a five-point Likert-type response scale, ranging from 1 (“almost never or never true”) to 5 (“almost always or always true”). The parent and peer forms assess three theoretically derived dimensions: Trust, Communication, and Alienation. Trust reflects perceived reliability, acceptance, and emotional availability in the attachment relationship. Communication reflects perceived openness and quality of communication with parents or peers. Alienation reflects feelings of anger, emotional distance, misunderstanding, or detachment within the relationship.

Illustrative items from the parent form include “I trust my parents” (Trust), “If my parents know something is bothering me, they ask me about it” (Communication), and “I get upset easily around my parents” (Alienation). Corresponding examples from the peer form include “I trust my friends” (Trust), “My friends care about how I am feeling” (Communication), and “I feel angry with my friends” (Alienation).

For the parent form, Trust includes 10 items, Communication includes 9 items, and Alienation includes 6 items. Accordingly, possible scores range from 10 to 50 for Parent Trust, 9 to 45 for Parent Communication, 6 to 30 for Parent Alienation, and 25 to 125 for Parent Total Security. For the peer form, Trust includes 10 items, Communication includes 8 items, and Alienation includes 7 items. Possible scores therefore range from 10 to 50 for Peer Trust, 8 to 40 for Peer Communication, 7 to 35 for Peer Alienation, and 25 to 125 for Peer Total Security.

Scoring followed the original IPPA item key (Armsden & Greenberg, 1987). Trust and Communication items were keyed so that higher scores indicated greater perceived attachment security. Negatively worded Trust and Communication items were reverse-coded as 6 minus the original response. Parent Trust items 3 and 9 and Parent Communication items 6 and 14 were reverse-coded. Peer Trust item 5 was reverse-coded, whereas none of the Peer Communication items required reverse coding.

Alienation items were retained in their original direction when calculating the Alienation subscales. Parent Alienation comprised items 8, 10, 11, 17, 18, and 23, while Peer Alienation comprised items 4, 9, 10, 11, 18, 22, and 23. No Alienation item was reverse-coded for the corresponding Alienation subscale. Higher Alienation scores therefore indicated greater emotional distance, anger, or detachment and lower perceived attachment security.

Parent Total Security and Peer Total Security were calculated after keying every item toward greater perceived security. Consequently, every Alienation item was reverse-coded when included in a Total Security score. For Parent Total Security, items 3, 6, 8, 9, 10, 11, 14, 17, 18, and 23 were reverse-coded. For Peer Total Security, items 4, 5, 9, 10, 11, 18, 22, and 23 were reverse-coded. Higher total scores therefore indicated greater perceived attachment security. Exact item membership, scoring direction, minimum completion thresholds, and scoring formulas are presented in Supplementary Table S1.

The Greek version was developed through a forward-translation, reconciliation, and back-translation procedure. Two Greek forward translations of the original English parent and peer forms were prepared separately, one by a child and adolescent psychiatrist and one by a professional translator. The two versions were subsequently compared item by item and reconciled into a single preliminary Greek version. Discrepancies were resolved by consensus, with priority given to conceptual equivalence, developmental appropriateness, and clarity for Greek-speaking adolescents rather than literal translation. Particular attention was given to preserving the intended meanings of trust, open communication, emotional distance, and alienation.

The reconciled Greek version was then back-translated into English by a native English speaker. The back-translated items were compared with the original English items, and discrepancies in meaning or emphasis were reviewed and resolved before the Greek version was finalized. The back-translator was not involved in preparing the forward translations; however, formal documentation of blinding to the original English version was not retained. Before the present psychometric study, the resulting Greek version had been used in routine child and adolescent psychiatric clinical practice for approximately three years. This period provided extended practical experience with the administration and wording of the instrument in Greek-speaking adolescents. However, this clinical use was not organized or recorded as a formal pilot study or cognitive-debriefing procedure, and no systematic qualitative data on item comprehension were collected. The present validation sample was recruited independently through participating schools and was not drawn from the clinical population in which the Greek IPPA had previously been used. The earlier clinical use formed part of routine care and did not constitute a study cohort or sampling frame for the present investigation. The IPPA authors currently state that no permission is required for its use or translation (Armsden & Greenberg, n.d.). Because Parent Trust item 9 and Peer Alienation item 9 showed anomalous item-level estimates in the present analyses, their exact finalized Greek wording and the English back-translations are provided in Supplementary Table S2 to facilitate evaluation of possible translation-related or semantic explanations.

#### 2.3.2. Strengths and Difficulties Questionnaire

The Strengths and Difficulties Questionnaire (SDQ) is a brief self-report screening instrument designed to assess emotional and behavioral adjustment in children and adolescents (Goodman, 1997, 2001). The adolescent self-report version was used in the present study. The SDQ includes 25 items rated on a three-point response scale: 0 (“not true”), 1 (“somewhat true”), and 2 (“certainly true”). Positively worded difficulty items are reverse-scored according to the standard SDQ scoring procedure.

The 25 items form five subscales, each consisting of five items: Emotional Symptoms, Conduct Problems, Hyperactivity/Inattention, Peer Problems, and Prosocial Behavior. Each subscale yields a score ranging from 0 to 10. The Emotional Symptoms subscale assesses worries, fears, unhappiness, somatic complaints, and nervousness. The Conduct Problems subscale assesses anger, obedience, fighting, lying, and stealing. The Hyperactivity/Inattention subscale assesses restlessness, fidgeting, distractibility, impulsivity, and persistence of attention. The Peer Problems subscale assesses loneliness, peer acceptance, bullying or victimization, friendships, and preference for adults. The Prosocial Behavior subscale assesses helping, sharing, kindness, and consideration for others. Representative items include “I worry a lot” (Emotional Symptoms), “I get very angry and often lose my temper” (Conduct Problems), “I am restless, I cannot stay still for long” (Hyperactivity/Inattention), “I am usually on my own” (Peer Problems), and “I try to be nice to other people” (Prosocial Behavior).

A Total Difficulties score is calculated by summing the Emotional Symptoms, Conduct Problems, Hyperactivity/Inattention, and Peer Problems subscales, yielding a possible range from 0 to 40. The Prosocial Behavior subscale is not included in the Total Difficulties score and is interpreted separately as a strengths-oriented index, with higher scores indicating more prosocial behavior.

The Greek version of the SDQ has previously been examined in a nationwide sample of Greek adolescents, with confirmatory factor analysis supporting the original five-factor structure after limited model modifications (Giannakopoulos et al., 2009). In that study, internal consistency was acceptable for Total Difficulties, Emotional Symptoms, and Prosocial Behavior, moderate for Hyperactivity/Inattention, and lower for Peer Problems and Conduct Problems. These findings support the use of the Greek SDQ as a brief community screening measure, while also indicating that individual subscales, particularly Peer Problems and Conduct Problems, should be interpreted cautiously.

In the present study, the SDQ was used as an external measure of adolescent psychosocial adjustment. We examined whether Parent Total Security and Peer Total Security showed the theoretically expected pattern of associations with the SDQ scales: negative associations with Emotional Symptoms, Conduct Problems, Hyperactivity/Inattention, Peer Problems, and Total Difficulties, and positive associations with Prosocial Behavior. Because the SDQ assesses emotional and behavioral adjustment rather than attachment, these correlations were interpreted as construct-validity evidence based on relations with external variables, not as convergent validity between measures of the same construct.

#### 2.3.3. Family Background and Sociodemographic Questionnaire

A separate family-background and sociodemographic form was completed by parents or legal guardians as part of the consent materials. Variables available in the analytic dataset included adolescent sex, school grade, birth year, parent-informant, family status, parent-informant educational level, parent-informant occupational status, subjective family economic status, perceived social class, and place of residence. Participating school site was obtained from the study administration records and coded anonymously as School Site 1 to School Site 5.

Parent-informant educational level and occupational status referred to the parent or legal guardian who completed the form and should not be interpreted as combined household-level indicators. Subjective family economic status and perceived social class were also parent-reported. Place of residence was used to describe the geographic distribution of the sample and to examine differences in residence composition across participating school sites. Analyses involving these variables were conducted using the available valid responses. Responses coded as unspecified or invalid were treated as missing.

### 2.4. Statistical Analysis

Data were inspected for invalid entries, out-of-range responses, and missing values before analysis. Response completion was evaluated at both the item and form levels, as described below. No dedicated careless-response index was applied. Invalid responses were treated as missing before scale computation. Reverse-coded IPPA and SDQ items were recoded according to their respective scoring keys. Scale scores were computed from valid item responses according to predefined scoring rules. An IPPA score was calculated only when at least 80% of the items belonging to the relevant scale had valid responses. Specifically, Parent Trust required at least 8 of 10 items, Parent Communication at least 8 of 9 items, Parent Alienation at least 5 of 6 items, and Parent Total Security at least 20 of 25 items. Peer Trust required at least 8 of 10 items, Peer Communication at least 7 of 8 items, Peer Alienation at least 6 of 7 items, and Peer Total Security at least 20 of 25 items.

When the completion threshold was met, the score was calculated as the mean of the available valid keyed item responses multiplied by the total number of items in the scale. Thus, Parent Total Security was calculated as the sum of the available valid parent items after security-direction keying, divided by the number of valid keyed parent items and multiplied by 25, provided that at least 20 parent items were valid. Peer Total Security was calculated using the corresponding peer-item formula and the same 20-item minimum requirement. Scores not meeting the corresponding completion threshold were treated as missing.

For the SDQ, each five-item subscale was computed when at least three valid items were available. When three or four items were valid, the subscale score was prorated as the mean of the available valid keyed item responses multiplied by five. Total Difficulties was computed as the sum of the four difficulty subscale scores. Therefore, analytic sample sizes vary slightly across scales and analyses.

The 80% completion rule applied to the observed IPPA scale scores; item-level WLSMV confirmatory analyses used available ordered-item responses as described below. Descriptive statistics included means, standard deviations, minimum and maximum values, skewness, and kurtosis. Floor and ceiling percentages were calculated as the proportions of participants with a valid observed score who attained the theoretical minimum or theoretical maximum, respectively. A floor or ceiling effect was considered notable when more than 15% of valid scores occurred at the corresponding endpoint (Terwee et al., 2007).

Item-level missingness was summarized separately for each parent and peer item using frequencies and percentages. Form-level completion status was defined among participants who had answered at least one item of the relevant form. Participants with valid responses to all 25 items were classified as complete responders, whereas those with one or more missing item responses were classified as incomplete responders. Complete and incomplete responders were compared on sex, grade, and participating school site using chi-square tests. They were also compared on SDQ Total Difficulties and the corresponding IPPA total score using Welch independent-samples *t*-tests. Comparisons involving an IPPA total score included only incomplete responders who met the 80% completion threshold. School-specific response rates were calculated from the numbers of eligible students invited and participating at each site and compared using a Pearson chi-square test, with Cramér’s V reported as the effect-size measure.

Data cleaning, descriptive statistics, group comparisons, correlations, and general linear models were conducted using IBM SPSS Statistics for Windows, version 31.0.0.0 (IBM Corp., Armonk, NY, USA). Confirmatory factor analyses were conducted in R version 4.5.0 using lavaan version 0.6-19. McDonald’s omega coefficients and bootstrap confidence intervals for the reliability coefficients were calculated in Python version 3.13.5 using scikit-learn version 1.8.0 and NumPy version 2.3.5.

Internal consistency of the IPPA scores was estimated using Cronbach’s alpha and McDonald’s omega total (Cronbach, 1951; McDonald, 1999). Reliability coefficients were interpreted as <0.70 low, 0.70–0.79 adequate, 0.80–0.89 good, and ≥0.90 excellent (Stefana et al., 2025). Corrected item-total correlations < 0.30 were considered weak. Standardized factor loadings with absolute values < 0.30 were likewise treated as weak item indicators (Stefana et al., 2025). Reliability coefficients for each IPPA score were calculated among participants with complete valid responses to all items contributing to that score. McDonald’s omega total was estimated from a one-factor maximum-likelihood common-factor model fitted to the standardized observed five-category item scores and was calculated as (Σλ)^2^/[(Σλ)^2^ + Σθ], where λ denotes the standardized factor loadings and θ denotes the item uniquenesses (McDonald, 1999). Because these reliability analyses were intended to characterize the internal consistency of the observed, unit-weighted IPPA scores reported in the study, omega was estimated from the observed item scores rather than from a polychoric correlation matrix. These values should therefore be interpreted as observed-score reliability estimates rather than as ordinal/polychoric reliability coefficients; the ordinal nature of the items was addressed separately in the structural analyses using WLSMV.

Nonparametric percentile 95% confidence intervals for alpha and omega were estimated using 2000 participant-level bootstrap resamples (Efron & Tibshirani, 1994). Cronbach’s alpha and its bootstrap confidence interval were additionally calculated for each SDQ subscale and for SDQ Total Difficulties among participants with complete valid responses to all items contributing to the relevant score. Classical item analysis of the IPPA was conducted using corrected item-total correlations and alpha-if-item-deleted coefficients.

Because the original three-factor structures of the parent and peer forms were specified a priori, confirmatory factor analysis was treated as the primary approach to structural validity. Confirmatory factor analyses were conducted separately for the parent and peer forms in R version 4.5.0 using lavaan version 0.6-19 (Rosseel, 2012). The complete-response ordinal sensitivity analyses were conducted in Python version 3.13.5 using NumPy version 2.3.5 and SciPy version 1.17.0. Because the IPPA items were rated on five ordered response categories, the items were treated as ordered categorical variables and models were estimated using the robust diagonally weighted least-squares estimator with mean- and variance-adjusted test statistics (WLSMV) (C.-H. Li, 2016; Xia & Yang, 2019).

Item-level missing data in the primary WLSMV confirmatory models were handled using pairwise-present observations, as implemented in lavaan. The parent analyses included 411 participants with at least one valid parent-form item, and the peer analyses included 409 participants with at least one valid peer-form item. Under pairwise-present estimation, participants with incomplete item data contributed only to the univariate and bivariate item information available from their observed responses; therefore, the nominal CFA sample sizes should not be interpreted as complete-case sample sizes. Effective pairwise sample sizes ranged from 385 to 411 for parent-item pairs and from 378 to 406 for peer-item pairs. These analytic sample sizes applied to the one-factor, two-factor, correlated three-factor, bifactor, and constrained factor-correlation models. The item-exclusion sensitivity analyses also retained *n* = 411 for the parent form and *n* = 409 for the peer form.

To assess the robustness of the primary structural findings to the inclusion of incomplete responders, the correlated three-factor structure was additionally re-estimated among participants with valid responses to all 25 items of the relevant form (parent, *n* = 319; peer, *n* = 320), using polychoric correlations and diagonally weighted least-squares estimation. This complete-response sensitivity analysis focused on the latent-factor correlation pattern, the loadings of the two previously identified anomalous items, and SRMR. No multigroup measurement-invariance models were retained in the revised ordinal analysis; consequently, there are no invariance-model analytic samples to report.

Sample-size planning was specified prospectively in the study protocol. The recruitment target was approximately 500 adolescents, with a sample of 350–400 considered the minimum for the main psychometric analyses, taking into account the planned factor analyses, internal-consistency estimation, and associations with the SDQ. The achieved total sample of 432 participants exceeded this predefined minimum, and the parent and peer CFA samples of 411 and 409 participants corresponded to more than 16 observations per item for each separately analyzed 25-item form. Sample-size requirements for structural equation and bifactor models are model-dependent and cannot be reduced to a single universal cutoff (Bader et al., 2022; Wolf et al., 2013). The bifactor models were therefore treated as secondary diagnostic analyses rather than as the primary tests of structural validity. Both bifactor models converged without improper residual variances, but their results were interpreted in conjunction with the correlated-factor models and bifactor-specific indices rather than as definitive evidence of dimensionality.

Given previous evidence that the factor structure of the IPPA, particularly the peer form, may vary across samples, one-factor, two-factor, and correlated three-factor models were compared for both forms (Maya et al., 2023; Pace et al., 2011; Tohme et al., 2024). A bifactor model was additionally examined to evaluate whether the item responses could be represented by a general perceived-security factor alongside narrower specific dimensions. Accordingly, four competing models were tested for each form: (a) a one-factor model in which all 25 items loaded on a single factor; (b) a two-factor model in which Trust and Communication items loaded on a combined Positive Relationship factor and Alienation items loaded on a separate factor; (c) the original correlated three-factor model comprising Trust, Communication, and Alienation; and (d) a bifactor model comprising a general perceived-security factor and orthogonal Trust, Communication, and Alienation specific factors. In the correlated-factor models, Alienation items were retained in their original direction. In the bifactor models, all items were keyed so that higher values indicated greater perceived attachment security.

Model fit was evaluated using the scaled χ^2^ statistic and the corresponding robust comparative fit index (CFI), Tucker–Lewis index (TLI), and root mean square error of approximation (RMSEA) with its 90% confidence interval, together with the standardized root mean square residual (SRMR). For descriptive interpretation, CFI and TLI values ≥ 0.90 and RMSEA and SRMR values ≤ 0.08 were used as broad reference points for at least acceptable fit, whereas CFI and TLI values ≥ 0.95, RMSEA ≤ 0.06, and SRMR ≤ 0.08 were used as more stringent reference points for close fit. These conventional values were used only as descriptive heuristics and not as fixed decision rules. The commonly cited thresholds were developed primarily for continuous indicators analyzed using maximum-likelihood estimation (L. Hu & Bentler, 1999), and simulation work has shown that CFI, TLI, and RMSEA may behave differently under DWLS/WLSMV estimation with ordered-categorical indicators (Xia & Yang, 2019). In particular, conventional cutoffs may provide an overly favorable impression of fit under categorical-data estimation. Accordingly, model fit in the present study was evaluated from the overall pattern of fit indices together with corrected nested-model comparisons, parameter estimates, factor correlations, and theoretical interpretability rather than by applying any single numerical cutoff as a pass/fail criterion.

Nested one-, two-, and three-factor WLSMV models were compared using lavaan’s lavTestLRT() function with the scaled-and-shifted Satorra (2000) difference test (method = “satorra.2000”, scaled.shifted = TRUE). This procedure applies the appropriate correction to the robust WLSMV test statistics; the reported difference-test statistics were therefore not obtained by simple subtraction of the individual model χ^2^ values. Because the bifactor models were not treated as directly nested alternatives, they were evaluated according to overall fit, convergence, residual variances, and the magnitude, direction, and statistical significance of the general and specific factor loadings.

To evaluate whether the unit-weighted total scores primarily represented a general perceived-security dimension, bifactor-derived indices were calculated following established recommendations (Rodriguez et al., 2016). These included model-based omega total (ωtotal), omega hierarchical (ωH), relative omega (ωH/ωtotal), explained common variance (ECV), and the percentage of uncontaminated correlations (PUC). Omega hierarchical estimates the proportion of total-score variance attributable uniquely to the general factor. Relative omega estimates the proportion of reliable total-score variance attributable to the general factor. ECV estimates the proportion of common variance explained by the general factor, whereas PUC represents the proportion of item correlations influenced only by the general factor. These indices were interpreted jointly with the magnitude and direction of the general and specific factor loadings. No single bifactor-derived index was treated as providing a definitive pass/fail threshold for dimensionality. Omega hierarchical, relative omega, ECV, and PUC were therefore interpreted jointly, with higher omega hierarchical, relative omega, and ECV values indicating greater general-factor saturation (Rodriguez et al., 2016).

Consistent with the a priori hypothesis-testing framework described by Wetzel and Killisch (2025), discriminant validity was examined using latent-factor correlations and their 95% confidence intervals. We expected the three latent dimensions to be statistically distinguishable from perfect positive or negative correlations, although substantial overlap, particularly between Trust and Communication, was anticipated. The freely estimated three-factor models were also compared with constrained models in which positive factor correlations were fixed to 1.00 or negative correlations involving Alienation were fixed to −1.00. The freely estimated and constrained factor-correlation models were compared using the same corrected scaled-and-shifted WLSMV χ^2^ difference-test procedure. Because correlations fixed at ±1 lie at the boundary of the admissible parameter space, these tests were interpreted jointly with the confidence intervals and the substantive magnitude of the correlations.

Standardized loadings from the correlated three-factor models are presented in Supplementary Table S2. Item-level sensitivity analyses were conducted by re-estimating the correlated three-factor parent model without Parent Trust item 9 and the correlated three-factor peer model without Peer Alienation item 9. These items were selected because Parent Trust item 9 had a loading statistically indistinguishable from zero, whereas Peer Alienation item 9 loaded significantly in the direction opposite to that expected. The reduced models were compared descriptively with the corresponding complete-item models using CFI, TLI, RMSEA with its 90% confidence interval, and SRMR.

Construct-validity evidence based on relations with external variables was examined using Pearson correlations of Parent Total Security and Peer Total Security with the SDQ indices. Nonparametric percentile 95% confidence intervals for the Pearson correlations were estimated using 2000 participant-level bootstrap resamples. Higher Parent Total Security and Peer Total Security scores were expected to correlate with fewer emotional and behavioral difficulties, fewer peer problems, lower total difficulties, and higher prosocial behavior. To provide a complete descriptive account of relations among the measured constructs, Pearson correlations were additionally calculated among all observed IPPA subscale and total scores and all SDQ subscale and Total Difficulties scores, using pairwise available valid scale scores. This complete correlation matrix was treated as descriptive and was not added to the prespecified multiplicity-adjusted test family.

Sex differences in observed IPPA scores were examined as exploratory descriptive comparisons using independent-samples tests and Cohen’s *d*. Formal measurement-invariance analyses across sex were not conducted. Given the unresolved structural concerns identified in the pooled-sample models, including substantial factor overlap and weaker structural performance of the peer form, multigroup ordinal invariance testing was deferred until the baseline measurement structure can be replicated more securely. For ordered-categorical indicators, invariance testing additionally requires explicit evaluation of threshold and loading invariance (Millsap & Yun-Tein, 2004; Putnick & Bornstein, 2016). Accordingly, the observed-score sex comparisons were treated as exploratory and were not interpreted as evidence of known-groups validity or latent mean differences (Chen, 2007; Putnick & Bornstein, 2016).

Differences in IPPA scores across participating school sites were examined using analysis of variance, Welch tests where appropriate (Welch, 1951), and general linear models adjusted for sex and grade. School-site differences in demographic and socioeconomic characteristics were examined using Pearson chi-square tests. To avoid sparse expected cell counts, family status was categorized as married versus other; place of residence as large city versus other; parent-informant educational level as secondary education or less, post-secondary training, or university degree; occupational status as employed versus not currently employed; subjective family economic status as well-off, average, or less well-off; and perceived social class as working, middle, or upper-middle/high. Employed status included full-time, part-time, occasional or casual, and self-employed work, while not-currently-employed status included unemployment, homemaking, student, retired or unable-to-work status, and other. Exploratory associations of parent-informant educational level, subjective family economic status, and perceived social class with Parent Total Security and Peer Total Security were examined using Spearman correlations. Subjective family economic status was oriented so that higher values indicated better perceived family economic circumstances. Occupational-status groups were compared using Welch independent-samples *t*-tests, mean differences with 95% confidence intervals, and Cohen’s *d* (Cohen, 1988; Welch, 1951). Analyses used available valid responses, and no imputation was performed. Multivariable socioeconomic regression models were not retained because these analyses were secondary and exploratory.

For descriptive interpretation of effect magnitude, absolute Pearson and Spearman correlation coefficients of approximately 0.10, 0.30, and 0.50 were considered small, medium, and large, respectively; absolute Cohen’s *d* values of approximately 0.20, 0.50, and 0.80 were considered small, medium, and large; and η^2^ values of approximately 0.01, 0.06, and 0.14 were interpreted as small, medium, and large, respectively (Cohen, 1988). These values were used as conventional descriptive benchmarks rather than rigid thresholds.

Because participants were nested within five participating school sites, school-level clustering was also evaluated descriptively. One-way random-effects intraclass correlation coefficients [ICC(1)] were calculated for Parent Total Security and Peer Total Security among participants with valid total-score and school-site data (McGraw & Wong, 1996). School site was represented as a categorical fixed factor in analyses that explicitly examined or adjusted for school-site differences. Multilevel confirmatory factor analysis was not conducted because only five level-2 units were available, which was insufficient for stable estimation of between-school variance and cluster-level standard errors (McNeish & Stapleton, 2016). The primary confirmatory factor models were therefore estimated at the individual level, and the possible effect of residual within-school dependence was considered when interpreting the results.

Multiplicity was addressed by distinguishing the hypothesis-driven psychometric analyses from the secondary exploratory analyses. The CFA model comparisons and reliability analyses addressed prespecified psychometric hypotheses and were not subjected to multiplicity adjustment. The 12 correlations between the two IPPA total scores and the six SDQ indices also addressed a prespecified construct-validity hypothesis and were therefore not classified as exploratory. Because multiple external-variable correlations were tested, the Benjamini–Hochberg false-discovery-rate procedure was nevertheless applied to these 12 correlations as a separate prespecified test family. The remaining exploratory analyses were grouped into four families: the eight observed-score sex comparisons; the six school-site omnibus and sensitivity tests comprising the standard ANOVA, Welch test, and sex- and grade-adjusted model for each IPPA total score; the nine school-site demographic and socioeconomic comparisons; and the eight associations between the four socioeconomic characteristics and the two IPPA total scores. Benjamini–Hochberg adjusted values are reported as *q*-values. Holm adjustment was retained for pairwise comparisons between participating school sites (Benjamini & Hochberg, 1995; Holm, 1979). All inferential tests were two-sided. Statistical significance was evaluated at *p* < 0.05 for unadjusted tests and *q* < 0.05 for analyses subject to false-discovery-rate adjustment.

Descriptive summaries of the observed Parent Total Security and Peer Total Security distributions were calculated using means, standard deviations, and the 25th, 50th, 75th, and 90th sample percentiles. Percentiles were estimated separately among participants with a valid total score using linear interpolation between adjacent ordered observations, corresponding to the Hyndman–Fan type 7 method (Hyndman & Fan, 1996). These summaries were intended only to describe the present sample and were not treated as normative reference values, clinical cutoffs, diagnostic thresholds, or screening thresholds.

## 3. Results

### 3.1. Missing Data, Descriptive Statistics, and Reliability

Item-level missingness was modest but was not identical across items. For the parent form, the number of missing responses per item ranged from 21 to 37, corresponding to 4.9% to 8.6% of the total sample. For the peer form, missing responses ranged from 24 to 41 per item, corresponding to 5.6% to 9.5%. Twenty-one participants had no valid parent-form item responses, and 23 had no valid peer-form item responses. Full item-level missingness results are presented in Supplementary Table S3.

Application of the 80% completion criterion yielded high usable-score rates across the IPPA scales, and very sparse responding was uncommon. Full item-level missingness and scale-specific completion results are presented in Supplementary Table S3.

Complete-versus-incomplete responder analyses showed no consistent demographic differences. Parent-form completion status differed by participating school site, whereas incomplete peer-form responders showed somewhat higher SDQ Total Difficulties and lower Peer Total Security. Full test statistics and effect sizes are reported in Supplementary Table S4.

Descriptive statistics and reliability estimates for all IPPA and SDQ scores are presented together in Table 2. Internal consistency was strongest for the IPPA total scores. Most IPPA subscales showed adequate-to-excellent reliability, whereas Peer Alienation was weaker. Among the SDQ scores, Total Difficulties showed good internal consistency, whereas Conduct Problems showed low internal consistency. Detailed reliability confidence intervals are reported in Supplementary Table S5.

No IPPA score exceeded the predefined 15% criterion for a notable floor or ceiling effect. Exact counts and percentages are reported in Supplementary Table S5.

### 3.2. Classical Item Analysis

Classical item analyses generally supported the internal coherence of the IPPA subscales, with two notable exceptions. Parent Trust item 9 showed a weak corrected item-total correlation and a negligible CFA loading, whereas Peer Alienation item 9 showed a near-zero corrected item-total correlation and a negative CFA loading. Deleting the latter item modestly improved its subscale internal consistency but did not resolve the broader structural concerns. Scale-level item-analysis results are summarized in Table 3, and full item-level CFA estimates are provided in Supplementary Table S2.

### 3.3. Confirmatory Factor Structure

Because the original three-factor structures of the parent and peer IPPA forms were specified a priori, confirmatory factor analysis was treated as the primary structural analysis. The CFA results are reported with reference to the reporting considerations summarized by Nye (2023), including the analytic sample, estimator, global model-fit indices, standardized parameter estimates, factor correlations, comparison of theoretically relevant alternative models, and transparency regarding sensitivity analyses and model modifications. All participants with at least one valid item response on the relevant IPPA form were retained for these analyses, yielding analytic samples of *n* = 411 for the parent form and *n* = 409 for the peer form. Confirmatory factor analyses were conducted using WLSMV estimation, treating the five-category item responses as ordered categorical variables. The correlated three-factor model showed acceptable fit for the parent form and weaker fit for the peer form. Latent-factor correlations were high in both forms and indicated limited discriminant validity, particularly between Peer Trust and Peer Communication. Complete model-fit statistics and factor correlations are presented in Table 4.

Standardized loadings were generally substantial, with two notable exceptions: Parent Trust item 9 had a weak, non-significant loading, whereas Peer Alienation item 9 loaded significantly in the direction opposite to that expected. Parent Trust item 3 showed an acceptable loading under the ordinal WLSMV model and was therefore not considered anomalous. Full standardized loadings, standard errors, confidence intervals, and *p*-values are presented in Supplementary Table S2.

The complete-response sensitivity analyses yielded the same substantive structural pattern. In the parent form (*n* = 319), the Trust–Communication, Trust–Alienation, and Communication–Alienation correlations were 0.96, −0.89, and −0.88, respectively, and SRMR was 0.05. Parent Trust item 9 remained weak (λ = 0.10). In the peer form (*n* = 320), the corresponding factor correlations were 0.98, −0.82, and −0.73, and SRMR was 0.07; Peer Alienation item 9 again loaded in the unexpected negative direction (λ = −0.22). These estimates were close in magnitude and direction to those from the primary pairwise-present analyses and preserved the same substantive conclusions, including the marked Trust–Communication overlap, particularly in the peer form, and the anomalous findings of the two identified items. Excluding incomplete responders therefore did not materially alter the structural interpretation.

### 3.4. Competing Factor Models and Discriminant Validity

For both the parent and peer forms, corrected scaled-and-shifted χ^2^ difference tests showed that the two-factor model improved on the one-factor model and that the correlated three-factor model improved further on the two-factor model. The correlated three-factor model therefore provided the best relative fit among the original one-, two-, and three-factor structures, although absolute fit remained weaker for the peer form. Full model-fit and nested-model comparison results are presented in Table 5.

Constrained-model tests indicated that all three latent factor pairs were statistically distinguishable in both forms. Nevertheless, the near-unity association between Peer Trust and Peer Communication indicated limited practical discriminant validity. Factor correlations and the corresponding constrained-model tests are summarized in Table 4.

The bifactor models showed lower approximate misfit indices than the corresponding correlated three-factor models, but these differences were not interpreted by themselves as evidence that the total scores were valid or preferable to the subscale scores. Bifactor-derived indices indicated substantial general-factor saturation, although several specific Trust-factor estimates showed limited interpretability. The bifactor models were therefore treated as secondary diagnostic analyses rather than as confirmatory evidence for either the total scores or the specific subscales. Full model-fit results are presented in Table 5, and detailed bifactor-derived indices are provided in Supplementary Table S6.

Sensitivity analyses excluding the two anomalous items did not materially improve the correlated three-factor models. Excluding Parent Trust item 9 produced no meaningful improvement in the parent model, while excluding Peer Alienation item 9 resulted in only minor changes in approximate fit indices and did not resolve the broader structural limitations of the peer form. Both items were therefore retained to preserve comparability with the original IPPA scoring structure, although the corresponding subscale results should be interpreted cautiously. No post hoc residual correlations or cross-loadings were introduced to improve model fit; the item-exclusion models were treated as sensitivity analyses rather than as revised confirmatory models. Full results from the item-exclusion sensitivity analyses are presented in Supplementary Table S7, and the primary competing models are presented in Table 5.

### 3.5. Associations Between IPPA and SDQ Scores

Reliability estimates for the SDQ scores are presented with the other descriptive and reliability statistics in Table 2. Because Conduct Problems showed low internal consistency and Peer Problems and Prosocial Behavior were less reliable than Total Difficulties, associations involving these subscales were interpreted more cautiously.

Higher Parent Total Security and Peer Total Security scores were associated with lower scores on all SDQ difficulty indices and higher Prosocial Behavior scores. The strongest association for Parent Total Security was with SDQ Total Difficulties, whereas the strongest association for Peer Total Security was with SDQ Peer Problems. All 12 IPPA–SDQ associations remained statistically significant after false-discovery-rate adjustment. Full correlation coefficients and 95% confidence intervals are presented in Table 6.

A complete observed-score correlation matrix, including all IPPA subscale and total scores and all SDQ subscale and Total Difficulties scores, is provided in Supplementary Table S8. These additional coefficients are presented descriptively. Correlations involving total scores and their component scales are mathematically non-independent because the corresponding scores are derived from overlapping items.

### 3.6. Exploratory Group Comparisons, Contextual Effects, and Descriptive Sample Percentiles

The participating school sites differed in residential composition, parent-informant educational level, and perceived social class after multiplicity adjustment. Associations between parent-reported socioeconomic characteristics and the IPPA total scores were generally weak, and none remained statistically significant after adjustment. Full demographic and socioeconomic analyses are reported in Supplementary Table S9.

Exploratory sex comparisons indicated higher Parent Alienation, Peer Total Security, Peer Trust, and Peer Communication among girls, with the largest observed difference in Peer Communication. Parent Trust, Parent Communication, Parent Total Security, and Peer Alienation did not differ significantly by sex. Because measurement invariance across sex was not examined, these findings remain descriptive and should not be interpreted as latent mean differences. Full results are presented in Supplementary Table S10.

School-level intraclass correlations were small. Nominal school-site differences in Parent Total Security and Peer Total Security did not remain statistically significant after false-discovery-rate adjustment and were therefore treated as exploratory rather than as robust contextual effects. Summary school-site results are presented in Supplementary Table S10.

The observed Parent Total Security and Peer Total Security percentiles are presented in Supplementary Table S10. These values describe the present convenience sample only and should not be interpreted as normative reference values.

## 4. Discussion

The present study provides an initial psychometric evaluation of the Greek IPPA in a school-based sample of adolescents aged 12 to 16 years. Three findings are central. First, Parent Total Security and Peer Total Security showed high internal consistency and were predominantly saturated by broad general factors. Second, although the correlated three-factor models performed better than simpler alternatives, the high latent correlations and poorly defined specific Trust factors indicated limited differentiation among the original subscales. Third, Parent Trust item 9 and Peer Alienation item 9 showed anomalous item-level results and require further investigation in the context of Greek cultural adaptation. The concurrent associations with the SDQ were theoretically coherent but do not establish criterion validity, temporal stability, or agreement with an independent attachment measure.

### 4.1. Broad Perceived-Security Scores and Limited Subscale Differentiation

The conventional reliability estimates indicated strong internal consistency, particularly for Parent Total Security and Peer Total Security. High alpha and omega coefficients, however, do not by themselves establish unidimensionality or demonstrate that the total scores are psychometrically preferable to the subscales (Rodriguez et al., 2016; Stefana et al., 2025). The bifactor-derived indices provided more direct information about the variance represented by the total scores and indicated substantial general-factor saturation, with most reliable total-score variance attributable to the corresponding general factors. These indices are most informative when considered jointly rather than as isolated decision thresholds, because substantial general-factor saturation can coexist with weak or poorly defined specific factors (Rodriguez et al., 2016). These findings provide preliminary support for interpreting the total scores as broad descriptive summaries of adolescents’ perceived relational security in the present sample.

The structural results nevertheless place important limits on subscale interpretation. The correlated three-factor models fitted significantly better than the corresponding one- and two-factor alternatives, and constrained-model comparisons indicated that the latent factors were statistically distinguishable. However, Trust and Communication were highly correlated in both forms, particularly in the peer form. For the peer form in particular, the statistically significant constrained-model test indicates a detectable departure from a perfect correlation but does not establish practically meaningful separation between Trust and Communication, given their near-unity estimated association (Wetzel & Killisch, 2025). Correlations involving Alienation were also high, particularly in the parent form. The better relative fit of the three-factor models therefore should not be equated with strong practical differentiation of Trust, Communication, and Alienation. The specific Trust factors in the bifactor models were also poorly defined, further limiting claims that the subscales capture clearly independent dimensions (Rodriguez et al., 2016).

This combination of better relative three-factor fit and high factor overlap is consistent with previous IPPA research. The original three-factor model has often performed better than simpler alternatives, but substantial correlations among its dimensions have also been reported (Demetriou et al., 2022; Pace et al., 2011). Structural difficulties have been particularly evident for the peer form, with some studies requiring model modification or item reduction and others failing to confirm the original peer structure (Habibi Asgarabad et al., 2025; Maya et al., 2023; Tohme et al., 2024). The present findings therefore add preliminary evidence from the Greek context to an already mixed international literature rather than establishing a definitive factor structure.

Within the present sample, Parent Total Security and Peer Total Security were more readily interpretable as broad descriptive summaries than the separate subscales, although this interpretation remains preliminary and requires independent replication (American Educational Research Association et al., 2014; Kane, 2013). Trust and Communication may still be reported as descriptive relational facets, but their high overlap means that differences between them should not receive strong substantive interpretation. Alienation requires a different directional interpretation because higher Alienation scores indicate greater emotional distance or detachment, whereas Alienation items are reverse-keyed when included in the total security scores. More generally, all IPPA scores describe adolescents’ self-reported perceptions of trust, communication, and alienation in their relationships. They do not identify attachment classifications, attachment disorders, attachment organization, or disorganization (Armsden & Greenberg, 1987; Madigan et al., 2016; Muzi et al., 2022).

### 4.2. Weak Items and Implications for Cultural Adaptation

The item-level analyses identified one problematic item in each form. Parent Trust item 9 showed a weak corrected item-total correlation and a negligible, non-significant CFA loading. Its removal did not materially improve global model fit, and the item was retained to preserve comparability with the original IPPA.

Peer Alienation item 9 showed a near-zero corrected item-total correlation and a standardized loading in the direction opposite to that expected. Removing the item modestly improved internal consistency and some approximate fit indices but did not resolve the broader structural limitations of the peer form. Immediate deletion would therefore be premature.

The content of Peer Alienation item 9 (direct English back-translation: “I feel the need to communicate with my friends more often”) may help explain its performance. A desire for more frequent contact with friends may reflect loneliness or unmet relational needs, but it may also reflect closeness, peer salience, social motivation, or contemporary expectations for frequent digital communication. Previous Greek-speaking and international research has similarly suggested that peer-contact items may be culturally and developmentally sensitive (Anbumalar & Binu Sahayam, 2026; Demetriou et al., 2022; Marini et al., 2025). Parent Trust item 9 (direct English back-translation: “My parents expect too much from me”) may likewise be affected by how adolescents interpret parental expectations in relation to support, pressure, achievement, or autonomy (Allen & Tan, 2016; Moretti & Peled, 2004). The exact finalized Greek wording of both items is provided in Supplementary Table S2.

The present results do not provide sufficient evidence to alter the established Greek scoring key. They do indicate that future cultural-adaptation work should move beyond translation and routine clinical use (International Test Commission, 2018). Structured cognitive interviews, adolescent feedback, qualitative examination of item meanings, and replication using item-response methods are needed to determine whether the anomalous findings arise from wording, cultural interpretation, developmental change, or sample-specific variation.

### 4.3. Relations with Psychosocial Adjustment and Exploratory Contextual Findings

Parent Total Security and Peer Total Security showed the expected pattern of concurrent associations with the SDQ. Adolescents reporting higher total security scores also reported fewer emotional and behavioral difficulties and more prosocial behavior. Parent Total Security showed a broad association with Total Difficulties, Emotional Symptoms, and Hyperactivity/Inattention, whereas Peer Total Security was particularly associated with Peer Problems. These patterns are consistent with previous evidence linking the quality of parent and peer relationships with adolescent emotional regulation, behavioral adjustment, peer functioning, and social competence (Brumariu & Kerns, 2010; Gorrese & Ruggieri, 2012; Madigan et al., 2016; Oldfield et al., 2016).

These correlations should be interpreted as theoretically coherent concurrent relations rather than as evidence of external or criterion validity. Both instruments were self-reported by the same adolescents during the same classroom assessment session, which may have increased the observed associations through shared method variance (Podsakoff et al., 2003). The SDQ is also a broad psychosocial measure rather than an attachment-specific comparator. In addition, the Conduct Problems subscale showed low internal consistency (α = 0.45), which substantially limits confidence in correlations involving this subscale (Giannakopoulos et al., 2009). Peer Problems and Prosocial Behavior also showed more modest reliability than Total Difficulties. Accordingly, associations involving SDQ Total Difficulties provide the strongest evidence, whereas correlations involving the less reliable SDQ subscales, particularly Conduct Problems, should be interpreted cautiously.

The sex comparisons were exploratory. Girls reported higher Parent Alienation, Peer Total Security, Peer Trust, and Peer Communication, with the largest difference found for Peer Communication. These observed-score differences require particular caution because measurement invariance across sex was not established. Meaningful interpretation of group differences requires evidence that the measurement structure operates equivalently across groups, including appropriate invariance of loadings and thresholds for ordered-categorical indicators (Chen, 2007; Millsap & Yun-Tein, 2004; Putnick & Bornstein, 2016).

We did not proceed to multigroup ordinal invariance testing because the pooled-sample structural analyses themselves identified unresolved measurement issues, including substantial Trust–Communication overlap, a near-unity Trust–Communication association in the peer form, and anomalous item-level estimates. We therefore considered it more appropriate to treat the sex comparisons as descriptive rather than to extend the current initial evaluation to group-specific latent comparisons before the baseline structure has been independently replicated (Putnick & Bornstein, 2016). Differences in self-disclosure, relational communication, or response style may also contribute to the observed-score pattern (Rose & Rudolph, 2006). Ordinal measurement invariance across sex remains an important priority for subsequent validation studies.

The school-site and socioeconomic analyses provided limited evidence for stable effects on the IPPA total scores. The participating schools differed in residential composition, parent-informant educational level, and perceived social class, but the school-site differences in Parent Total Security and Peer Total Security did not remain statistically significant after false-discovery-rate adjustment. The socioeconomic associations with the IPPA total scores were small, and none remained statistically significant after adjustment across the eight tests. The largest nominal association was between better subjective family economic status and Parent Total Security. These exploratory results do not provide evidence of a robust or independent socioeconomic effect and should be interpreted as sample-specific.

The reported percentiles are similarly restricted to descriptive use. They summarize the observed total-score distributions in the present convenience sample but do not constitute population norms, diagnostic thresholds, clinical cutoffs, or screening thresholds. Representative normative work would require predefined probability-based sampling and evidence that the scores function equivalently across relevant demographic groups (American Educational Research Association et al., 2014; Kane, 2013).

### 4.4. Clinical and Research Implications

The present findings support the use of the Greek IPPA primarily for research and descriptive group-level purposes in samples similar to the present school-based sample. Parent Total Security and Peer Total Security may be interpreted as broad descriptive summaries of adolescents’ self-reported perceptions of relational security at the group level, whereas the separate subscales, particularly Trust and Communication, require greater caution because of their substantial overlap. The current evidence does not support using IPPA scores for individual clinical decision-making. More generally, the intended interpretation and use of test scores should not extend beyond the validity evidence available for those interpretations and uses (American Educational Research Association et al., 2014; Kane, 2013).

The IPPA should not be used as a stand-alone screening or diagnostic instrument to identify attachment classifications, attachment disorders, or disorganization, or to guide treatment or other individual-level decisions. Before individual-level interpretation could be considered, stronger evidence would be required regarding content validity, including cognitive interviewing, test–retest reliability, measurement invariance across relevant groups, representative reference data, and convergence with an independent attachment-specific assessment (American Educational Research Association et al., 2014; International Test Commission, 2018). In clinical contexts, if the IPPA is administered, its scores should be treated only as descriptive self-report information about perceived relationship quality and interpreted alongside, rather than as a substitute for, a comprehensive assessment. This restriction is consistent with the distinction between questionnaire measures of perceived relationship quality and interview- or observation-based assessments of attachment organization (Madigan et al., 2016; Muzi et al., 2022).

### 4.5. Limitations and Future Directions

Several sampling, administration, and measurement limitations constrain interpretation of the findings. The five participating schools formed a non-probability convenience sample from Attica, and the overall response rate was 40.6%. Response rates varied significantly across the five participating school sites, ranging from 29.6% to 58.7%, indicating differential participation across schools. Because no demographic or psychosocial data were available for non-participants, however, the presence and direction of individual-level participation bias could not be determined (Turk et al., 2018). Selective participation could have affected the observed score distributions, psychometric estimates, or associations if participating adolescents and families differed systematically from those who did not participate. The sample was also predominantly urban, and parent-informants were relatively highly educated, limiting generalizability to adolescents from rural settings and families with lower educational or socioeconomic representation.

Item-level missingness was generally modest, but incomplete peer-form responders had somewhat higher SDQ difficulties and lower Peer Total Security, suggesting that peer-form nonresponse was not entirely random. Although the achieved CFA sample sizes supported estimation of the planned models, the bifactor findings remain secondary and should be replicated in larger independent samples (Bader et al., 2022; Rodriguez et al., 2016).

The participants were nested within only five schools. This number was insufficient for stable multilevel CFA, and the item-level WLSMV models were therefore estimated at the individual level without cluster-robust standard errors or another model-based adjustment for school clustering. Although the observed-score intraclass correlations were small, residual within-school dependence may still have affected parameter standard errors and model-fit statistics. Replication should include substantially more schools and use cluster-adjusted or multilevel ordinal CFA (McNeish & Stapleton, 2016).

Questionnaire administration was conducted under conditions that protected response privacy; no fixed time limit was imposed, and members of the research team were available to answer questions. Nevertheless, no dedicated careless-response or response-pattern index was applied. Data were checked for invalid entries, out-of-range responses, and missingness, but undetected inattentive responding cannot be completely excluded.

The cross-sectional, self-report design prevents conclusions about temporal stability, prediction, or causality and may have increased shared method variance (Podsakoff et al., 2003). No attachment-specific questionnaire, interview, or observational measure was included, so convergence with an independent assessment of attachment-related representations could not be evaluated (Madigan et al., 2016; Muzi et al., 2022).

Test–retest reliability was not assessed, and ordinal measurement invariance was not examined across sex, age, grade, school site, or socioeconomic groups. Consequently, equivalence of item thresholds and factor loadings across these groups cannot be assumed, and observed-score group differences should not be interpreted as latent group differences (Millsap & Yun-Tein, 2004; Putnick & Bornstein, 2016). Measurement invariance across sex is an important priority for subsequent validation, irrespective of whether direct sex comparisons are undertaken. Invariance across age, grade, school site, and socioeconomic groups should also be examined in sufficiently large and well-represented samples.

The parent form used a plural, non-specific parent referent and did not record whether an adolescent answered with both parents or primarily one parent in mind. Parent-form scores may therefore reflect somewhat different relational referents across participants, particularly across different family structures.

The conventional alpha and omega estimates also have an estimator-related limitation. They were calculated from the observed five-category item scores rather than from a polychoric correlation matrix and should therefore be interpreted as observed-score reliability estimates rather than as ordinal/polychoric reliability coefficients. Although the structural analyses treated the items as ordered categorical variables using WLSMV, future studies should also examine ordinal reliability estimates explicitly.

The translation procedure included forward translation, reconciliation, and back-translation, but it did not include a formally documented cognitive-debriefing study. The back-translator’s blinding was not documented. These limitations are particularly relevant to the anomalous item-level results for Parent Trust item 9 and Peer Alienation item 9 (International Test Commission, 2018).

Future studies should replicate the structural models in larger and more diverse Greek samples, include enough schools for cluster-adjusted analyses, examine ordinal measurement invariance, and assess test–retest reliability. Longitudinal and clinical studies should evaluate predictive relations and compare IPPA scores with attachment-specific interviews or other relational measures. Formal cognitive interviewing with Greek adolescents should focus particularly on parental expectations, peer contact, online communication, and the meaning of relational closeness before any item modification is recommended.

## 5. Conclusions

The present study provides initial psychometric evidence concerning the Greek IPPA in a school-based adolescent sample. Parent Total Security and Peer Total Security showed high internal consistency and substantial general-factor saturation, providing preliminary, sample-specific support for research and descriptive use at the group level, but not for individual clinical decision-making. The correlated three-factor models performed better than simpler alternatives, but the high latent correlations and poorly defined specific Trust factors provided limited support for independent interpretation of Trust and Communication. Parent Trust item 9 and Peer Alienation item 9 require further cultural and developmental investigation. The theoretically coherent associations with SDQ scores provide evidence of relations with concurrent psychosocial adjustment but do not establish criterion validity or causality. IPPA scores do not identify attachment classifications, attachment disorders, or disorganization, and the reported percentiles should not be interpreted as norms, clinical cutoffs, or screening thresholds. Replication in larger and more diverse samples, together with evidence on test–retest reliability, measurement invariance, attachment-specific comparisons, and cognitive interviewing, is required.

## Figures and Tables

**Table 1:** Sample characteristics.

Characteristic	*n* (%) or *M* (*SD*), Range
Total sample	432 (100.0)
Age, years	13.95 (0.85), 12–16
Adolescent sex	
Girls	212 (49.9)
Boys	213 (50.1)
School grade	
1st Gymnasium grade	163 (38.2)
2nd Gymnasium grade	145 (34.0)
3rd Gymnasium grade	119 (27.9)
Participating school site	
School Site 1	89 (20.6)
School Site 2	111 (25.7)
School Site 3	75 (17.4)
School Site 4	80 (18.5)
School Site 5	77 (17.8)
Parent-informant	
Mother	351 (83.0)
Father	72 (17.0)
Family status	
Married parents	337 (79.7)
Widowed parent	10 (2.4)
Divorced parents	57 (13.5)
Separated parents	13 (3.1)
Unmarried parents	6 (1.4)
Place of residence	
Large city	318 (75.0)
Suburbs	104 (24.5)
Town or small city	2 (0.5)
Parent-informant educational level	
Primary school graduate	2 (0.5)
Lower secondary school graduate	3 (0.7)
Upper secondary school graduate	83 (19.9)
Post-secondary training	155 (37.1)
University degree	175 (41.9)
Parent-informant occupational status	
Employed full-time	347 (82.8)
Employed part-time	37 (8.8)
Occasionally or casually employed	4 (1.0)
Unemployed or looking for work	7 (1.7)
Homemaker	17 (4.1)
Student, retired, or unable to work due to illness/disability	6 (1.4)
Other	1 (0.2)
Subjective family economic status	
Very well-off	8 (1.9)
Quite well-off	63 (15.1)
Average	315 (75.5)
Not very well-off	26 (6.2)
Not at all well-off	5 (1.2)
Perceived social class	
Working class	53 (12.7)
Middle class	275 (65.9)
Upper-middle class	87 (20.9)
High class	2 (0.5)

Note. Percentages are based on valid responses for each variable. Valid age data were available for 422 participants, sex data for 425 participants, grade data for 427 participants, school-site data for 432 participants, birth-year data for 422 participants, parent-informant and family-status data for 423 participants, and place-of-residence data for 424 participants. Parent-informant educational level was available for 418 participants, occupational status for 419 participants, and subjective family economic status and perceived social class for 417 participants each. School sites are anonymized for confidentiality reasons and should not be interpreted as Directorate-level categories.

**Table 2:** Descriptive statistics and reliability estimates for IPPA and SDQ scores.

Scale	*n*	*M*	*SD*	Observed Range	Skewness	Kurtosis	α (*n*)	ω (*n*)
IPPA scores								
Parent Trust	410	41.82	5.87	15–50	−1.39	2.20	0.82 (357)	0.85 (357)
Parent Communication	408	34.29	6.97	10–45	−0.71	0.17	0.87 (390)	0.87 (390)
Parent Alienation	408	13.91	4.86	6–29	0.53	−0.17	0.80 (377)	0.81 (377)
Parent Total Security	409	98.13	16.15	37–125	−0.96	0.70	0.94 (319)	0.94 (319)
Peer Trust	406	42.58	6.46	11–50	−1.32	2.40	0.89 (376)	0.90 (376)
Peer Communication	405	31.49	5.79	10–40	−0.60	0.04	0.87 (380)	0.87 (380)
Peer Alienation	400	16.28	4.46	7–31	0.58	0.04	0.71 (365)	0.74 (365)
Peer Total Security	407	99.83	14.70	35–124	−0.90	1.11	0.93 (320)	0.94 (320)
SDQ scores								
Emotional Symptoms	411	3.10	2.62	0–10	0.76	−0.28	0.77 (403)	—
Conduct Problems	411	2.82	1.70	0–9	0.85	0.59	0.45 (404)	—
Hyperactivity/Inattention	411	3.73	2.58	0–10	0.39	−0.68	0.73 (403)	—
Peer Problems	411	1.70	1.91	0–9	1.42	1.75	0.69 (403)	—
Prosocial Behavior	411	8.05	1.78	2–10	−0.78	−0.07	0.61 (407)	—
Total Difficulties	411	11.35	6.35	0–33	0.69	−0.06	0.82 (387)	—

Note. IPPA = Inventory of Parent and Peer Attachment; SDQ = Strengths and Difficulties Questionnaire; α = Cronbach’s alpha; ω = McDonald’s omega. For the IPPA scores, *n* denotes participants meeting the 80% completion threshold for the corresponding observed score. For the SDQ, each five-item subscale was calculated when at least three valid items were available, and Total Difficulties was calculated from the four difficulty subscales. The *n* values in parentheses following the reliability coefficients denote participants with complete valid responses to all items contributing to the corresponding reliability estimate. McDonald’s omega was estimated for the IPPA scores only. Observed range refers to the minimum and maximum values observed in the analytic sample. Higher IPPA Alienation scores indicate greater alienation and lower attachment security. Reliability confidence intervals and IPPA floor and ceiling percentages are reported in Supplementary Table S5.

**Table 3:** Item analysis summary.

Scale	Corrected Item-Total Range	Main Interpretation
Parent Trust	0.09 to 0.70	Variable corrected item-total correlations; one weak item
Parent Communication	0.44 to 0.76	Acceptable to strong corrected item-total correlations
Parent Alienation	0.44 to 0.65	Acceptable to strong corrected item-total correlations
Peer Trust	0.53 to 0.70	Strong corrected item-total correlations
Peer Communication	0.45 to 0.70	Acceptable to strong corrected item-total correlations
Peer Alienation	0.01 to 0.64	Variable corrected item-total correlations; one item had a near-zero item-total correlation and a negative CFA loading

Note. Corrected item-total correlations were calculated within each corresponding IPPA subscale. The lowest corrected item-total correlation was observed for a Peer Alienation item referring to the need for more frequent contact with friends. This item also showed a statistically significant negative loading in the WLSMV confirmatory model. Parent Trust variability was mainly due to one item referring to parents expecting too much.

**Table 4:** Correlated three-factor confirmatory model results for the parent and peer IPPA forms.

Analysis	Parent IPPA	Peer IPPA
CFA estimator	WLSMV	WLSMV
CFA analytic *n*	411	409
CFA model	Correlated three-factor	Correlated three-factor
χ^2^(*df*)	602.99 (272)	941.90 (272)
CFI	0.97	0.94
TLI	0.97	0.93
RMSEA (90% CI)	0.05 (0.05–0.06)	0.08 (0.07–0.08)
SRMR	0.05	0.07
Trust–Communication *r* (95% CI)	0.93 (0.91–0.95)	0.98 (0.96–0.99)
Trust–Alienation *r* (95% CI)	−0.88 (−0.91 to −0.84)	−0.82 (−0.87 to −0.78)
Communication–Alienation *r* (95% CI)	−0.85 (−0.89 to −0.80)	−0.71 (−0.78 to −0.64)
Trust–Communication constraint, Δχ^2^(*df*), *p*	40.41 (1), <0.001	7.10 (1), 0.008
Trust–Alienation constraint, Δχ^2^(*df*), *p*	42.46 (1), <0.001	65.82 (1), <0.001
Communication–Alienation constraint, Δχ^2^(*df*), *p*	57.72 (1), <0.001	71.28 (1), <0.001
Interpretation	Best relative original-factor model; high factor overlap	Best relative original-factor model; weaker fit and near-unity Trust–Communication correlation

Note. IPPA = Inventory of Parent and Peer Attachment; CFA = confirmatory factor analysis; WLSMV = weighted least-squares mean- and variance-adjusted estimator; CFI = comparative fit index; TLI = Tucker–Lewis index; RMSEA = root mean square error of approximation; SRMR = standardized root mean square residual. CFA indices are scaled WLSMV fit statistics. Negative correlations with Alienation are expected because Alienation was retained in its original direction, with higher scores indicating greater alienation and lower perceived attachment security. The parent and peer constrained factor-correlation models used the same analytic samples as the corresponding freely estimated models: *n* = 411 and *n* = 409, respectively. The constrained factor-correlation models were compared with the corresponding freely estimated models using the corrected scaled-and-shifted Satorra (2000) χ^2^ difference test implemented in lavTestLRT(); the reported Δχ^2^ values are not simple differences between the individual model χ^2^ statistics.

**Table 5:** Competing WLSMV CFA models for the parent and peer IPPA forms.

Model	χ^2^	*df*	CFI	TLI	RMSEA (90% CI)	SRMR	Scaled Δχ^2^(*df*), *p* vs. Preceding Nested Model	Interpretation
Parent form (*n* = 411)								
One-factor	734.03	275	0.96	0.96	0.06 (0.06–0.07)	0.06	—	Inferior to the two- and three-factor models
Two-factor: Positive Relationship and Alienation	645.43	274	0.97	0.96	0.06 (0.05–0.06)	0.05	50.55 (1), <0.001	Improved over the one-factor model
Correlated three-factor	602.99	272	0.97	0.97	0.05 (0.05–0.06)	0.05	41.39 (2), <0.001	Best original-factor model; high factor overlap
Bifactor	507.27	250	0.98	0.97	0.05 (0.04–0.06)	0.04	—	Lower approximate misfit indices; substantial general-factor saturation but unstable Trust-specific loadings
Peer form (*n* = 409)								
One-factor	1137.19	275	0.92	0.91	0.09 (0.08–0.09)	0.07	—	Weakest peer model
Two-factor: Positive Relationship and Alienation	970.55	274	0.93	0.93	0.08 (0.07–0.08)	0.07	79.79 (1), <0.001	Improved over the one-factor model
Correlated three-factor	941.90	272	0.94	0.93	0.08 (0.07–0.08)	0.07	32.61 (2), <0.001	Best original-factor model; near-unity Trust–Communication correlation
Bifactor	769.86	250	0.95	0.94	0.07 (0.07–0.08)	0.06	—	Lower approximate misfit indices; substantial general-factor saturation but unstable Trust-specific loadings

Note. IPPA = Inventory of Parent and Peer Attachment; WLSMV = weighted least-squares mean- and variance-adjusted estimator; CFA = confirmatory factor analysis; CFI = comparative fit index; TLI = Tucker–Lewis index; RMSEA = root mean square error of approximation; SRMR = standardized root mean square residual. The nested one-, two-, and correlated three-factor models were compared using the corrected scaled-and-shifted Satorra (2000) χ^2^ difference test implemented in lavaan’s lavTestLRT() (method = “satorra.2000”, scaled.shifted = TRUE); the reported Δχ^2^ values are not simple differences between the individual model χ^2^ statistics. The bifactor models were evaluated from overall fit and the interpretability of their general and specific factor loadings.

**Table 6:** Correlations between IPPA total security scores and SDQ scores.

SDQ Scale	Parent Total Security, *r* (95% CI)	Peer Total Security, *r* (95% CI)
Emotional Symptoms	−0.44 [−0.52, −0.36]	−0.30 [−0.40, −0.20]
Conduct Problems	−0.37 [−0.47, −0.27]	−0.23 [−0.32, −0.14]
Hyperactivity/Inattention	−0.49 [−0.56, −0.41]	−0.31 [−0.39, −0.22]
Peer Problems	−0.38 [−0.47, −0.28]	−0.49 [−0.58, −0.39]
Prosocial Behavior	0.22 [0.13, 0.32]	0.28 [0.18, 0.38]
Total Difficulties	−0.59 [−0.66, −0.53]	−0.46 [−0.54, −0.37]

Note. SDQ = Strengths and Difficulties Questionnaire; IPPA = Inventory of Parent and Peer Attachment; CI = confidence interval. Correlations used SDQ subscale scores calculated when at least three of the five relevant items were valid. Parent Total Security correlations included 409 participants, and Peer Total Security correlations included 407 participants. Confidence intervals for Pearson correlations are nonparametric percentile bootstrap intervals based on 2000 participant-level resamples. Benjamini–Hochberg adjustment was applied across the 12 IPPA–SDQ correlations; all associations remained statistically significant at *q* < 0.001. All unadjusted correlations were statistically significant at *p* < 0.001. Reliability estimates are reported in Table 2, with confidence intervals in Supplementary Table S5.

## Data Availability

The data presented in this study are not publicly available due to privacy and ethical restrictions. De-identified data may be made available from the corresponding author upon reasonable request, subject to institutional approval and compliance with applicable data protection regulations.

## Supplementary Materials

The following supporting information can be downloaded at: https://www.mdpi.com/article/10.3390/ejihpe16090134/s1, Table S1: Item composition, scoring direction, and computation of the Greek IPPA scores; Table S2: Standardized loadings from the correlated three-factor WLSMV models for the Greek IPPA parent and peer forms and wording of the two items with anomalous item-level estimates; Table S3: Item-level missingness and scale-specific completion thresholds for the Greek IPPA; Table S4: Comparisons between complete and incomplete IPPA form responders; Table S5: Reliability confidence intervals for the Greek IPPA and SDQ scores and floor and ceiling effects for the Greek IPPA scores; Table S6: Bifactor-derived reliability and dimensionality indices for the Greek IPPA parent and peer total scores; Table S7: Sensitivity analyses excluding the anomalous Parent Trust and Peer Alienation items; Table S8: Pearson correlations among all observed IPPA and SDQ scores; Table S9: Exploratory school-site demographic comparisons and associations of parent-reported socioeconomic characteristics with IPPA total scores; Table S10: Exploratory group comparisons, contextual effects, and descriptive sample percentiles.

## Abbreviations

The following abbreviations are used in this manuscript:

CFA	Confirmatory factor analysis
CFI	Comparative fit index
ECV	Explained common variance
IPPA	Inventory of Parent and Peer Attachment
PUC	Percentage of uncontaminated correlations
RMSEA	Root mean square error of approximation
SRMR	Standardized root mean square residual
SDQ	Strengths and Difficulties Questionnaire
TLI	Tucker–Lewis index
WLSMV	Weighted least-squares mean- and variance-adjusted estimator
