# Supplementary material for: Initial Psychometric Evaluation of the Inventory of Parent and Peer Attachment in Greek Adolescents
Source: Eur J Investig Health Psychol Educ. 2026 Sep 2;16(9):134. doi: 10.3390/ejihpe16090134 (PMC13605670; doi:10.3390/ejihpe16090134)
Supplement: Supplementary file 1 [file ejihpe-16-00134-s001.zip › Table S1.pdf]

Table S1. Item composition, scoring direction, and computation of the Greek IPPA scores.

Score	Direct-keyed items	Reverse-keyed items	Minimum valid items	Computation	Higher values indicate
Parent Trust	1, 2, 4, 12, 13, 20, 21, 22	3, 9	8 of 10	Mean of valid keyed items × 10	Greater trust
Parent Communication	5, 7, 15, 16, 19, 24, 25	6, 14	8 of 9	Mean of valid keyed items × 9	Better communication
Parent Alienation	8, 10, 11, 17, 18, 23	None	5 of 6	Mean of valid original-direction items × 6	Greater alienation and lower security
Parent Total Security	1, 2, 4, 5, 7, 12, 13, 15, 16, 19, 20, 21, 22, 24, 25	3, 6, 8, 9, 10, 11, 14, 17, 18, 23	20 of 25	Mean of valid security-keyed items × 25	Greater perceived parent relational security
Peer Trust	6, 8, 12, 13, 14, 15, 19, 20, 21	5	8 of 10	Mean of valid keyed items × 10	Greater trust
Peer Communication	1, 2, 3, 7, 16, 17, 24, 25	None	7 of 8	Mean of valid items × 8	Better communication
Peer Alienation	4, 9, 10, 11, 18, 22, 23	None	6 of 7	Mean of valid original-direction items × 7	Greater alienation and lower security
Peer Total Security	1, 2, 3, 6, 7, 8, 12, 13, 14, 15, 16, 17, 19, 20, 21, 24, 25	4, 5, 9, 10, 11, 18, 22, 23	20 of 25	Mean of valid security-keyed items × 25	Greater perceived peer relational security

Note. Scoring followed the original IPPA item key (Armsden & Greenberg, 1987). Items were rated from 1 to 5. For reverse-keyed items, the recoded response was calculated as 6 minus the original response. Alienation items were retained in their original direction when calculating the Alienation subscales. Consequently, higher Alienation subscale scores indicate greater alienation and lower perceived attachment security. In contrast, every Alienation item was reverse-coded when calculating Parent Total Security or Peer Total Security. A score was calculated only when the stated minimum number of valid items was available. When this threshold was met, the score was calculated as the mean of the valid keyed item responses multiplied by the total number of items in the corresponding scale. Item numbers refer to the original item order within each 25-item form. Parent Total Security = mean[P1, P2, (6 − P3), P4, P5, (6 − P6), P7, (6 − P8), (6 − P9), (6 − P10), (6 − P11), P12, P13, (6 − P14), P15, P16, (6 − P17), (6 − P18), P19, P20, P21, P22, (6 − P23), P24, P25] × 25, provided that at least 20 valid items are available. Peer Total Security = mean[Pe1, Pe2, Pe3, (6 − Pe4), (6 − Pe5), Pe6, Pe7, Pe8, (6 − Pe9), (6 − Pe10), (6 − Pe11), Pe12, Pe13, Pe14, Pe15, Pe16, Pe17, (6 − Pe18), Pe19, Pe20, Pe21, (6 − Pe22), (6 − Pe23), Pe24, Pe25] × 25, provided that at least 20 valid items are available. P = parent-form item; Pe = peer-form item.
