# Supplementary material for: Initial Psychometric Evaluation of the Inventory of Parent and Peer Attachment in Greek Adolescents
Source: Eur J Investig Health Psychol Educ. 2026 Sep 2;16(9):134. doi: 10.3390/ejihpe16090134 (PMC13605670; doi:10.3390/ejihpe16090134)
Supplement: Supplementary file 1 [file ejihpe-16-00134-s001.zip › Table S10.pdf]

Table S10. Exploratory group comparisons, contextual effects, and descriptive sample percentiles.

Domain	Main result	Interpretation
Sex, Parent Trust	$p = 0.133$, $q = 0.152$, $d = -0.15$	No significant difference
Sex, Parent Communication	$p = 0.702$, $q = 0.702$, $d = -0.04$	No significant difference
Sex, Parent Alienation	$p < 0.001$, $q = 0.001$, $d = 0.35$	Higher observed Alienation among girls
Sex, Parent Total Security	$p = 0.084$, $q = 0.134$, $d = -0.17$	No significant difference
Sex, Peer Trust	$p = 0.001$, $q = 0.002$, $d = 0.33$	Higher observed score among girls
Sex, Peer Communication	$p < 0.001$, $q < 0.001$, $d = 0.65$	Higher observed score among girls; medium effect
Sex, Peer Alienation	$p = 0.132$, $q = 0.152$, $d = 0.15$	No significant difference
Sex, Peer Total Security	$p < 0.001$, $q = 0.001$, $d = 0.36$	Higher observed score among girls
Participating school site, Parent Total Security	$F(4,404) = 2.49$, $p = 0.043$, $q = 0.054$, $\eta^2 = 0.02$; ICC = 0.02	Small nominal variation; did not survive FDR adjustment
Participating school site, Peer Total Security	$F(4,402) = 2.68$, $p = 0.031$, $q = 0.054$, $\eta^2 = 0.03$; ICC = 0.02	Small nominal variation; did not survive FDR adjustment
Parent-reported socioeconomic characteristics	Largest nominal association: subjective family economic status with Parent Total Security, $\varrho = 0.13$, $p = 0.010$, $q = 0.078$	No socioeconomic association remained significant after FDR adjustment
Parent Total Security distribution	$M = 98.13$, $SD = 16.15$, P25 = 89.58, P50 = 101.00, P75 = 110.00, P90 = 115.30	Descriptive sample percentiles; $n = 409$
Peer Total Security distribution	$M = 99.83$, $SD = 14.70$, P25 = 91.00, P50 = 102.00, P75 = 111.00, P90 = 116.80	Descriptive sample percentiles; $n = 407$

Note. All IPPA scores were calculated using the 80% completion rule. Sex comparisons are exploratory observed-score comparisons and should not be interpreted as evidence of known-groups validity or latent mean differences. ICC = intraclass correlation coefficient. The ICCs were estimated descriptively using one-way random-effects analyses among participants with valid total-score and school-site data: Parent Total Security, $n = 409$; Peer Total Security, $n = 407$. Because only five schools participated, the ICCs and school-site comparisons are sample-specific and should not be interpreted as population-level school effects. *P*25, *P*50, *P*75, and *P*90 denote the 25th, 50th, 75th, and 90th sample percentiles, respectively. Percentiles were calculated using linear interpolation between adjacent ordered observations among participants with valid total scores: Parent Total Security, $n = 409$; Peer Total Security, $n = 407$. The distribution summaries describe the present convenience sample only and should not be interpreted as normative reference values, clinical cutoffs, or screening thresholds. Participating school-site effects refer to differences between anonymized school sites, not to differences between Directorates of Secondary Education. *q*-values were calculated using the Benjamini–Hochberg false-discovery-rate procedure within the prespecified exploratory test families.
