# Supplementary material for: Initial Psychometric Evaluation of the Inventory of Parent and Peer Attachment in Greek Adolescents
Source: Eur J Investig Health Psychol Educ. 2026 Sep 2;16(9):134. doi: 10.3390/ejihpe16090134 (PMC13605670; doi:10.3390/ejihpe16090134)
Supplement: Supplementary file 1 [file ejihpe-16-00134-s001.zip › Table S2.pdf]

Table S2. Standardized loadings from the correlated three-factor WLSMV models for the Greek IPPA parent and peer forms and wording of the two items with anomalous item-level estimates.

Panel A. Standardized loadings from the correlated three-factor WLSMV models for the Greek IPPA parent and peer forms.

Form	Factor	Item	Reversed	Standardized loading	*SE*	95% CI	*p*
Parent	Trust	1	No	0.78	0.03	[0.72, 0.83]	< 0.001
Parent	Trust	2	No	0.67	0.04	[0.59, 0.74]	< 0.001
Parent	Trust	4	No	0.68	0.04	[0.60, 0.75]	< 0.001
Parent	Trust	12	No	0.77	0.03	[0.72, 0.83]	< 0.001
Parent	Trust	13	No	0.64	0.03	[0.57, 0.71]	< 0.001
Parent	Trust	20	No	0.86	0.02	[0.82, 0.90]	< 0.001
Parent	Trust	21	No	0.81	0.02	[0.77, 0.85]	< 0.001
Parent	Trust	22	No	0.80	0.03	[0.74, 0.85]	< 0.001
Parent	Trust	3	Yes	0.56	0.05	[0.46, 0.66]	< 0.001
Parent	Trust	9	Yes	0.09	0.05	[−0.02, 0.19]	0.097
Parent	Communication	5	No	0.64	0.03	[0.58, 0.70]	< 0.001
Parent	Communication	7	No	0.50	0.04	[0.41, 0.58]	< 0.001
Parent	Communication	15	No	0.80	0.02	[0.76, 0.84]	< 0.001
Parent	Communication	16	No	0.79	0.02	[0.75, 0.84]	< 0.001
Parent	Communication	19	No	0.83	0.02	[0.80, 0.87]	< 0.001
Parent	Communication	24	No	0.88	0.02	[0.84, 0.91]	< 0.001
Parent	Communication	25	No	0.66	0.04	[0.59, 0.73]	< 0.001
Parent	Communication	6	Yes	0.73	0.03	[0.68, 0.78]	< 0.001
Parent	Communication	14	Yes	0.53	0.04	[0.45, 0.60]	< 0.001
Parent	Alienation	8	No	0.68	0.04	[0.61, 0.75]	< 0.001
Parent	Alienation	10	No	0.76	0.03	[0.71, 0.80]	< 0.001
Parent	Alienation	11	No	0.71	0.03	[0.65, 0.77]	< 0.001
Parent	Alienation	17	No	0.65	0.03	[0.58, 0.71]	< 0.001
Parent	Alienation	18	No	0.69	0.04	[0.62, 0.76]	< 0.001
Parent	Alienation	23	No	0.65	0.04	[0.58, 0.73]	< 0.001
Peer	Trust	6	No	0.78	0.02	[0.74, 0.82]	< 0.001
Peer	Trust	8	No	0.69	0.03	[0.63, 0.76]	< 0.001

Form	Factor	Item	Reversed	Standardized loading	*SE*	95% CI	*p*
Peer	Trust	12	No	0.81	0.02	[0.77, 0.85]	< 0.001
Peer	Trust	13	No	0.82	0.03	[0.77, 0.87]	< 0.001
Peer	Trust	14	No	0.69	0.03	[0.63, 0.75]	< 0.001
Peer	Trust	15	No	0.73	0.03	[0.69, 0.78]	< 0.001
Peer	Trust	19	No	0.79	0.02	[0.75, 0.83]	< 0.001
Peer	Trust	20	No	0.80	0.02	[0.76, 0.84]	< 0.001
Peer	Trust	21	No	0.80	0.02	[0.76, 0.84]	< 0.001
Peer	Trust	5	Yes	0.73	0.04	[0.66, 0.80]	< 0.001
Peer	Communication	1	No	0.55	0.04	[0.48, 0.62]	< 0.001
Peer	Communication	2	No	0.74	0.02	[0.70, 0.79]	< 0.001
Peer	Communication	3	No	0.77	0.02	[0.72, 0.82]	< 0.001
Peer	Communication	7	No	0.74	0.03	[0.69, 0.80]	< 0.001
Peer	Communication	16	No	0.69	0.03	[0.64, 0.75]	< 0.001
Peer	Communication	17	No	0.79	0.02	[0.74, 0.84]	< 0.001
Peer	Communication	24	No	0.80	0.02	[0.76, 0.85]	< 0.001
Peer	Communication	25	No	0.71	0.03	[0.66, 0.77]	< 0.001
Peer	Alienation	4	No	0.65	0.04	[0.57, 0.72]	< 0.001
Peer	Alienation	9	No	−0.23	0.06	[−0.35, −0.12]	< 0.001
Peer	Alienation	10	No	0.52	0.05	[0.43, 0.62]	< 0.001
Peer	Alienation	11	No	0.79	0.03	[0.72, 0.86]	< 0.001
Peer	Alienation	18	No	0.54	0.05	[0.46, 0.63]	< 0.001
Peer	Alienation	22	No	0.67	0.03	[0.61, 0.74]	< 0.001
Peer	Alienation	23	No	0.74	0.03	[0.68, 0.80]	< 0.001

Note. Values are standardized loadings from the correlated three-factor WLSMV models, with the five-category item responses treated as ordered variables. Items marked as reversed were reverse-coded before analysis so that higher values indicated greater Trust or Communication. Alienation items were retained in their original direction, with higher values indicating greater alienation and lower perceived attachment security. Parent Trust item 9 was the only loading statistically indistinguishable from zero, $\lambda = 0.09$, 95% CI [−0.02, 0.19], $p = 0.097$. Peer Alienation item 9 loaded significantly in the direction opposite to that expected, $\lambda = -0.23$, 95% CI [−0.35, −0.12], $p < 0.001$. Parent Trust item 3 showed an acceptable and statistically significant loading under WLSMV estimation, $\lambda = 0.56$, 95% CI [0.46, 0.66], $p < 0.001$. Item numbers refer to the original item order within each 25-item IPPA form. The parent model included $n = 411$ participants, and the peer model included $n = 409$ participants.

Panel B. Final Greek wording and direct English back-translation of the two items with anomalous item-level estimates

Item	Final Greek wording	Direct English back-translation
Parent Trust item 9	Οι γονείς μου περιμένουν πάρα πολλά από εμένα.	My parents expect too much from me.
Peer Alienation item 9	Νιώθω την ανάγκη να επικοινωνώ πιο συχνά με τους φίλους μου.	I feel the need to communicate with my friends more often.

Note. The English wording in Panel B is the back-translation of the finalized Greek wording, provided to facilitate evaluation of possible translation-related or semantic explanations for the anomalous item-level findings.
