# Supplementary material for: Initial Psychometric Evaluation of the Inventory of Parent and Peer Attachment in Greek Adolescents
Source: Eur J Investig Health Psychol Educ. 2026 Sep 2;16(9):134. doi: 10.3390/ejihpe16090134 (PMC13605670; doi:10.3390/ejihpe16090134)
Supplement: Supplementary file 1 [file ejihpe-16-00134-s001.zip › Table S3.pdf]

Table S3. Item-level missingness and scale-specific completion thresholds for the Greek IPPA.

Panel A. Item-level missingness.

Form	Item	Missing *n*	Missing %
Parent	1	22	5.1
Parent	2	22	5.1
Parent	3	29	6.7
Parent	4	37	8.6
Parent	5	24	5.6
Parent	6	25	5.8
Parent	7	25	5.8
Parent	8	24	5.6
Parent	9	25	5.8
Parent	10	29	6.7
Parent	11	29	6.7
Parent	12	27	6.2
Parent	13	32	7.4
Parent	14	25	5.8
Parent	15	24	5.6
Parent	16	26	6.0
Parent	17	27	6.2
Parent	18	25	5.8
Parent	19	21	4.9
Parent	20	23	5.3
Parent	21	30	6.9
Parent	22	28	6.5
Parent	23	31	7.2
Parent	24	22	5.1
Parent	25	21	4.9
Peer	1	26	6.0
Peer	2	27	6.2
Peer	3	31	7.2
Peer	4	27	6.2

**Form**	**Item**	**Missing *n***	**Missing %**
Peer	5	26	6.0
Peer	6	30	6.9
Peer	7	39	9.0
Peer	8	27	6.2
Peer	9	37	8.6
Peer	10	34	7.9
Peer	11	28	6.5
Peer	12	31	7.2
Peer	13	31	7.2
Peer	14	28	6.5
Peer	15	26	6.0
Peer	16	27	6.2
Peer	17	26	6.0
Peer	18	41	9.5
Peer	19	30	6.9
Peer	20	28	6.5
Peer	21	29	6.7
Peer	22	28	6.5
Peer	23	27	6.2
Peer	24	24	5.6
Peer	25	26	6.0

Panel B. Scale-specific completion.

Scale	Total items	Minimum valid items	Participants meeting threshold, *n*	% of total sample
Parent Trust	10	8	410	94.9
Parent Communication	9	8	408	94.4
Parent Alienation	6	5	408	94.4
Parent Total Security	25	20	409	94.7
Peer Trust	10	8	406	94.0
Peer Communication	8	7	405	93.8
Peer Alienation	7	6	400	92.6
Peer Total Security	25	20	407	94.2

Note. Percentages are based on the full sample of 432 adolescents. Missing responses include absent and out-of-range item responses. A score was calculated only when at least 80% of the items belonging to the relevant scale had valid responses.
