# Supplementary material for: Initial Psychometric Evaluation of the Inventory of Parent and Peer Attachment in Greek Adolescents
Source: Eur J Investig Health Psychol Educ. 2026 Sep 2;16(9):134. doi: 10.3390/ejihpe16090134 (PMC13605670; doi:10.3390/ejihpe16090134)
Supplement: Supplementary file 1 [file ejihpe-16-00134-s001.zip › Table S4.pdf]

Table S4. Comparisons between complete and incomplete IPPA form responders.

Form	Comparison	Complete responders	Incomplete responders	Test result
Parent	Sex distribution	$n = 315$	$n = 92$	$\chi^2(1) = 2.29$, $p = 0.130$
Parent	Grade distribution	$n = 316$	$n = 92$	$\chi^2(2) = 2.39$, $p = 0.303$
Parent	School-site distribution	$n = 319$	$n = 92$	$\chi^2(4) = 11.79$, $p = 0.019$
Parent	SDQ Total Difficulties, *M* (*SD*)	11.07 (6.26), $n = 319$	12.36 (6.61), $n = 92$	Welch $t(141.49) = -1.67$, $p = 0.097$, $d = -0.20$
Parent	Parent Total Security, *M* (*SD*)	98.33 (16.52), $n = 319$	97.45 (14.85), $n = 90$	Welch $t(156.66) = 0.49$, $p = 0.628$, $d = 0.05$
Peer	Sex distribution	$n = 316$	$n = 88$	$\chi^2(1) = 3.27$, $p = 0.071$
Peer	Grade distribution	$n = 317$	$n = 88$	$\chi^2(2) = 5.47$, $p = 0.065$
Peer	School-site distribution	$n = 320$	$n = 89$	$\chi^2(4) = 6.16$, $p = 0.188$
Peer	SDQ Total Difficulties, *M* (*SD*)	10.96 (6.14), $n = 320$	12.75 (6.97), $n = 88$	Welch $t(126.53) = -2.19$, $p = 0.030$, $d = -0.28$
Peer	Peer Total Security, *M* (*SD*)	100.69 (14.81), $n = 320$	96.65 (13.88), $n = 87$	Welch $t(143.77) = 2.37$, $p = 0.019$, $d = 0.28$

Note. Complete responders provided valid responses to all 25 items of the relevant form. Incomplete responders provided at least one valid item response but had one or more missing responses. IPPA total-score comparisons included only incomplete responders who met the 80% completion threshold. Positive Cohen's *d* values indicate higher scores among complete responders; negative values indicate higher scores among incomplete responders.
