# Supplementary material for: Initial Psychometric Evaluation of the Inventory of Parent and Peer Attachment in Greek Adolescents
Source: Eur J Investig Health Psychol Educ. 2026 Sep 2;16(9):134. doi: 10.3390/ejihpe16090134 (PMC13605670; doi:10.3390/ejihpe16090134)
Supplement: Supplementary file 1 [file ejihpe-16-00134-s001.zip › Table S5.pdf]

Table S5. Reliability confidence intervals for the Greek IPPA and SDQ scores and floor and ceiling effects for the Greek IPPA scores.

Panel A. Greek IPPA scores.

Scale	Score *n*	Reliability *n*	Cronbach's α (95% CI)	McDonald's ω (95% CI)	Floor, *n* (%)	Ceiling, *n* (%)
Parent Trust	410	357	0.82 [0.78, 0.85]	0.85 [0.81, 0.88]	0 (0.0)	10 (2.4)
Parent Communication	408	390	0.87 [0.85, 0.89]	0.87 [0.85, 0.89]	0 (0.0)	14 (3.4)
Parent Alienation	408	377	0.80 [0.77, 0.83]	0.81 [0.77, 0.84]	17 (4.2)	0 (0.0)
Parent Total Security	409	319	0.94 [0.92, 0.95]	0.94 [0.93, 0.95]	0 (0.0)	2 (0.5)
Peer Trust	406	376	0.89 [0.87, 0.91]	0.90 [0.87, 0.92]	0 (0.0)	46 (11.3)
Peer Communication	405	380	0.87 [0.85, 0.89]	0.87 [0.85, 0.89]	0 (0.0)	23 (5.7)
Peer Alienation	400	365	0.71 [0.66, 0.75]	0.74 [0.69, 0.77]	3 (0.8)	0 (0.0)
Peer Total Security	407	320	0.93 [0.91, 0.94]	0.94 [0.92, 0.95]	0 (0.0)	0 (0.0)

Panel B. Strengths and Difficulties Questionnaire scores.

SDQ score	Reliability *n*	Cronbach's α (95% CI)
Emotional Symptoms	403	0.77 [0.72, 0.80]
Conduct Problems	404	0.45 [0.35, 0.53]
Hyperactivity/Inattention	403	0.73 [0.68, 0.77]
Peer Problems	403	0.69 [0.62, 0.74]
Prosocial Behavior	407	0.61 [0.54, 0.67]
Total Difficulties	387	0.82 [0.79, 0.84]

Note. CI = confidence interval; IPPA = Inventory of Parent and Peer Attachment; SDQ = Strengths and Difficulties Questionnaire. Score *n* denotes the number of participants meeting the 80% completion threshold for the corresponding observed IPPA score. Reliability *n* denotes participants with complete valid responses to every item contributing to the relevant score. McDonald's omega total was estimated from a one-factor maximum-likelihood common-factor model fitted to standardized item responses and calculated as $(\Sigma\lambda)^2/[(\Sigma\lambda)^2 + \Sigma\theta]$, where λ denotes standardized factor loadings and θ denotes item uniquenesses. These omega coefficients are observed-score reliability estimates based on the original five-category item scores and are not polychoric/ordinal reliability coefficients. Confidence intervals for alpha and omega are nonparametric percentile bootstrap intervals based on 2,000 participant-level resamples. Floor and ceiling percentages are the proportions attaining the theoretical minimum and maximum, respectively, among participants with a valid observed score. A floor or ceiling effect was considered notable when more than 15% of valid scores occurred at the corresponding theoretical endpoint. No IPPA score exceeded this criterion.
