# Supplementary material for: Initial Psychometric Evaluation of the Inventory of Parent and Peer Attachment in Greek Adolescents
Source: Eur J Investig Health Psychol Educ. 2026 Sep 2;16(9):134. doi: 10.3390/ejihpe16090134 (PMC13605670; doi:10.3390/ejihpe16090134)
Supplement: Supplementary file 1 [file ejihpe-16-00134-s001.zip › Table S6.pdf]

Table S6. Bifactor-derived reliability and dimensionality indices for the Greek IPPA parent and peer total scores.

Form	Model-based ωtotal	ωH	Relative ω	ECV	PUC	General-factor loading range	Interpretation
Parent	0.96	0.93	0.97	0.85	0.68	0.07 to 0.86	Substantial general-factor saturation; preliminary support for total-score interpretation
Peer	0.96	0.92	0.96	0.83	0.69	−0.25 to 0.82	Substantial general-factor saturation; preliminary support for total-score interpretation, with one anomalous item

Note. Estimates were derived from the orthogonal bifactor WLSMV models using standardized factor loadings and residual variances. ωtotal = model-based omega total; ωH = omega hierarchical; Relative ω = ωH/ωtotal; ECV = explained common variance; PUC = percentage of uncontaminated correlations. Relative omega represents the proportion of reliable total-score variance attributable to the general factor. The negative lower bound of the peer general-factor loading range reflects Peer Alienation item 9, which loaded in the opposite direction from the remaining security-keyed items. The parent bifactor model included n = 411 participants, and the peer bifactor model included n = 409 participants. No single bifactor-derived index was treated as a definitive pass/fail threshold; the indices were interpreted jointly. The resulting support for total-score interpretation is preliminary and sample-specific and is restricted to broad descriptive use at the group level, not individual-level interpretation.
