# Supplementary material for: Initial Psychometric Evaluation of the Inventory of Parent and Peer Attachment in Greek Adolescents
Source: Eur J Investig Health Psychol Educ. 2026 Sep 2;16(9):134. doi: 10.3390/ejihpe16090134 (PMC13605670; doi:10.3390/ejihpe16090134)
Supplement: Supplementary file 1 [file ejihpe-16-00134-s001.zip › Table S7.pdf]

Table S7. Sensitivity analyses excluding the anomalous Parent Trust and Peer Alienation items.

Form	Model	*n*	χ^2 (*df*)	CFI	TLI	RMSEA (90% CI)	SRMR	Interpretation
Parent	Primary 25-item correlated three-factor model	411	602.99 (272)	0.97	0.97	0.05 (0.05–0.06)	0.05	Primary model
Parent	Model excluding Parent Trust item 9	411	568.41 (249)	0.97	0.97	0.06 (0.05–0.06)	0.05	No material improvement
Peer	Primary 25-item correlated three-factor model	409	941.90 (272)	0.94	0.93	0.08 (0.07–0.08)	0.07	Primary model
Peer	Model excluding Peer Alienation item 9	409	864.24 (249)	0.94	0.93	0.08 (0.07–0.08)	0.06	Minor changes in approximate fit indices; broader misfit remained

Note. CFI = comparative fit index; TLI = Tucker–Lewis index; RMSEA = root mean square error of approximation; SRMR = standardized root mean square residual. All models were estimated using WLSMV with the item responses treated as ordered categorical variables. Because the full-item and reduced-item models contained different observed items, differences in χ^2 were not interpreted as formal nested-model tests. The sensitivity analyses were evaluated descriptively from changes in the approximate fit indices. Excluding the anomalous item did not alter the analytic sample size for either form. No post hoc residual correlations or cross-loadings were introduced; the item-exclusion models were treated as sensitivity analyses rather than as revised confirmatory models.
