# Supplementary material for: Initial Psychometric Evaluation of the Inventory of Parent and Peer Attachment in Greek Adolescents
Source: Eur J Investig Health Psychol Educ. 2026 Sep 2;16(9):134. doi: 10.3390/ejihpe16090134 (PMC13605670; doi:10.3390/ejihpe16090134)
Supplement: Supplementary file 1 [file ejihpe-16-00134-s001.zip › Table S8.pdf]

Table S8. Pearson correlations among all observed IPPA and SDQ scores.

Score	1	2	3	4	5	6	7	8	9	10	11	12	13	14
1. Parent Trust	—													
2. Parent Communication	0.79*	—												
3. Parent Alienation	−0.72*	−0.72*	—											
4. Parent Total Security	0.92*	0.94*	−0.87*	—										
5. Peer Trust	0.30*	0.32*	−0.33*	0.35*	—									
6. Peer Communication	0.25*	0.30*	−0.24*	0.29*	0.84*	—								
7. Peer Alienation	−0.36*	−0.38*	0.59*	−0.47*	−0.60*	−0.47*	—							
8. Peer Total Security	0.34*	0.37*	−0.41*	0.41*	0.95*	0.90*	−0.75*	—						
9. Emotional Symptoms	−0.36*	−0.34*	0.56*	−0.44*	−0.25*	−0.12*	0.48*	−0.30*	—					
10. Conduct Problems	−0.32*	−0.32*	0.38*	−0.37*	−0.22*	−0.17*	0.21*	−0.23*	0.21*	—				
11. Hyperactivity/Inattention	−0.43*	−0.44*	0.48*	−0.49*	−0.28*	−0.21*	0.36*	−0.31*	0.39*	0.37*	—			
12. Peer Problems	−0.32*	−0.32*	0.40*	−0.38*	−0.47*	−0.36*	0.47*	−0.49*	0.47*	0.24*	0.36*	—		
13. Prosocial Behavior	0.20*	0.26*	−0.11*	0.22*	0.25*	0.37*	−0.08	0.28*	0.11*	−0.30*	−0.25*	−0.13*	—	
14. Total Difficulties	−0.51*	−0.50*	0.65*	−0.59*	−0.42*	−0.29*	0.54*	−0.46*	0.77*	0.58*	0.78*	0.70*	−0.17*	—

IPPA = Inventory of Parent and Peer Attachment; SDQ = Strengths and Difficulties Questionnaire. Values are Pearson correlation coefficients calculated using pairwise available valid scale scores; pairwise analytic sample sizes ranged from 397 to 411. An asterisk indicates statistical significance at the unadjusted two-tailed $p < 0.05$ level; no multiplicity adjustment was applied to this descriptive correlation matrix. Alienation scores were retained in their original direction, such that higher Parent Alienation and Peer Alienation scores indicate greater alienation and lower perceived attachment security. The matrix is presented descriptively to provide the complete pattern of associations among the measured constructs. Parent Total Security and Peer Total Security are computed from the corresponding IPPA items, and SDQ Total Difficulties is computed from the four difficulty subscales; correlations involving these composite scores and their component scales are therefore

mathematically non-independent and should be interpreted descriptively. The 12 prespecified correlations of Parent Total Security and Peer Total Security with the six SDQ scores, including their 95% confidence intervals and multiplicity-adjusted inference, are reported in Table 6 of the main manuscript.
