# Supplementary material for: Initial Psychometric Evaluation of the Inventory of Parent and Peer Attachment in Greek Adolescents
Source: Eur J Investig Health Psychol Educ. 2026 Sep 2;16(9):134. doi: 10.3390/ejihpe16090134 (PMC13605670; doi:10.3390/ejihpe16090134)
Supplement: Supplementary file 1 [file ejihpe-16-00134-s001.zip › Table S9.pdf]

Table S9. Exploratory school-site demographic comparisons and associations of parent-reported socioeconomic characteristics with IPPA total scores.

Panel A. School-site comparisons of demographic and socioeconomic characteristics.

Characteristic	Categories used for comparison	*n*	$\chi^2(df)$	*p*	Benjamini–Hochberg *q*
Adolescent sex	Girls; boys	425	1.09 (4)	0.895	0.895
School grade	Grades 1, 2, and 3	427	11.90 (8)	0.156	0.234
Parent-informant	Mother; father	423	3.85 (4)	0.427	0.548
Family status	Married parents; other	423	2.78 (4)	0.595	0.669
Place of residence	Large city; other	424	50.79 (4)	< 0.001	<0.001
Parent-informant educational level	Secondary education or less; post-secondary training; university degree	418	31.72 (8)	< 0.001	<0.001
Parent-informant occupational status	Employed; not currently employed	419	6.79 (4)	0.148	0.234
Subjective family economic status	Well-off; average; less well-off	417	13.47 (8)	0.097	0.218
Perceived social class	Working class; middle class; upper-middle/high class	417	47.10 (8)	< 0.001	<0.001

Panel B. Exploratory associations with Parent Total Security and Peer Total Security.

Socioeconomic characteristic	Outcome	Analytic *n*	Effect estimate (95% CI)	*p*	Benjamini–Hochberg *q*
Parent-informant educational level	Parent Total Security	401	Spearman $\varrho = -0.06$ [−0.15, 0.04]	0.263	0.516
Parent-informant educational level	Peer Total Security	399	Spearman $\varrho = -0.08$ [−0.17, 0.02]	0.119	0.474
Parent-informant occupational status	Parent Total Security	402	$\Delta M = 3.33$ [−3.64, 10.31]; $d = 0.21$	0.338	0.516
Parent-informant occupational status	Peer Total Security	400	$\Delta M = 0.69$ [−4.72, 6.09]; $d = 0.05$	0.798	0.798
Subjective family economic status	Parent Total Security	400	Spearman $\varrho = 0.13$ [0.03, 0.22]	0.010	0.078
Subjective family economic status	Peer Total Security	398	Spearman $\varrho = 0.02$ [−0.08, 0.12]	0.708	0.798
Perceived social class	Parent Total Security	400	Spearman $\varrho = 0.04$ [−0.05, 0.14]	0.387	0.516
Perceived social class	Peer Total Security	398	Spearman $\varrho = -0.05$ [−0.14, 0.05]	0.350	0.516

Note. IPPA = Inventory of Parent and Peer Attachment; CI = confidence interval; ΔM = mean difference. For Panel A, categories with small frequencies were combined before the chi-square analyses: family status was coded as married versus other; residence as large city versus other; educational level as secondary education or less, post-secondary training, or university degree; occupational status as employed versus not currently employed; subjective economic status as well-off, average, or less well-off; and social class as working, middle, or upper-middle/high. Employed included full-time, part-time, occasional/casual, and self-employed work; not currently employed included unemployment, homemaking, student/retired/unable-to-work status, and other. Analytic *n* in Panel A reflects participants with valid data for the characteristic being compared. In Panel B, education, subjective economic status, and social class were analyzed as ordered variables using Spearman correlations. Subjective economic status was oriented so that higher values indicated better perceived family economic circumstances. Occupational-status effects compare employed with not currently employed participants using Welch tests; positive ΔM and *d* values indicate higher IPPA scores in the employed group. Correlation confidence intervals are nonparametric percentile bootstrap intervals based on 2,000 participant-level resamples. Benjamini–Hochberg adjustment was applied separately across the nine school-site comparisons in Panel A and the eight socioeconomic association tests in Panel B. No association in Panel B remained statistically significant after false-discovery-rate adjustment.
